# Dissecting human embryonic skeletal stem cell ontogeny by single-cell transcriptomic and functional analyses

**DOI:** 10.1038/s41422-021-00467-z

**Published:** 2021-01-20

**Authors:** Jian He, Jing Yan, Jianfang Wang, Liangyu Zhao, Qian Xin, Yang Zeng, Yuxi Sun, Han Zhang, Zhijie Bai, Zongcheng Li, Yanli Ni, Yandong Gong, Yunqiao Li, Han He, Zhilei Bian, Yu Lan, Chunyu Ma, Lihong Bian, Heng Zhu, Bing Liu, Rui Yue

**Affiliations:** 1 State Key Laboratory of Proteomics, Academy of Military Medical Sciences, Academy of Military Sciences, Beijing, 100071 China; 2 Institute for Regenerative Medicine, Shanghai East Hospital, Shanghai Key Laboratory of Signaling and Disease Research, Frontier Science Center for Stem Cell Research, School of Life Sciences and Technology, Tongji University, Shanghai, 200092 China; 3 Department of Orthopedics, Changzheng Hospital, Naval Medical University, Shanghai, 200003 China; 4 State Key Laboratory of Experimental Hematology, Fifth Medical Center of Chinese PLA General Hospital, Beijing, 100071 China; 5 Department of Cardiology, Shanghai Tenth People’s Hospital, Tongji University School of Medicine, Shanghai, 200072 China; 6 Department of Transfusion, Daping Hospital, Army Military Medical University, Chongqing, 400042 China; 7 Department of Hematology, The First Affiliated Hospital of Zhengzhou University, Zhengzhou, Henan 450052 China; 8 Key Laboratory for Regenerative Medicine of Ministry of Education, Institute of Hematology, School of Medicine, Jinan University, Guangzhou, Guangdong 510632 China; 9 Guangzhou Regenerative Medicine and Health-Guangdong Laboratory (GRMH-GDL), Guangzhou, Guangdong 510530 China; 10 Department of Gynecology, Fifth Medical Center of Chinese PLA General Hospital, Beijing, 100071 China; 11 Beijing Institute of Radiation Medicine, Beijing, 100850 China

**Keywords:** Mesenchymal stem cells, Transcriptomics

## Abstract

Human skeletal stem cells (SSCs) have been discovered in fetal and adult long bones. However, the spatiotemporal ontogeny of human embryonic SSCs during early skeletogenesis remains elusive. Here we map the transcriptional landscape of human limb buds and embryonic long bones at single-cell resolution to address this fundamental question. We found remarkable heterogeneity within human limb bud mesenchyme and epithelium, and aligned them along the proximal–distal and anterior–posterior axes using known marker genes. Osteo-chondrogenic progenitors first appeared in the core limb bud mesenchyme, which give rise to multiple populations of stem/progenitor cells in embryonic long bones undergoing endochondral ossification. Importantly, a perichondrial embryonic skeletal stem/progenitor cell (eSSPC) subset was identified, which could self-renew and generate the osteochondral lineage cells, but not adipocytes or hematopoietic stroma. eSSPCs are marked by the adhesion molecule CADM1 and highly enriched with FOXP1/2 transcriptional network. Interestingly, neural crest-derived cells with similar phenotypic markers and transcriptional networks were also found in the sagittal suture of human embryonic calvaria. Taken together, this study revealed the cellular heterogeneity and lineage hierarchy during human embryonic skeletogenesis, and identified distinct skeletal stem/progenitor cells that orchestrate endochondral and intramembranous ossification.

## Introduction

Multipotent and self-renewing skeletal stem cells (SSCs) were identified in the growth plate of early postnatal mice by prospective isolation and genetic lineage-tracing studies.^1,2^ SSCs were also found within PTHrP^+^ chondrocytes in the resting zone of mouse postnatal growth plate,^3^ and in the periosteum of postnatal long bones and calvaria (also known as periosteal stem cells, PSCs).^4^ Importantly, human SSCs were recently identified in the growth plate of 17-week-old fetal long bones, suggesting that SSCs exist in higher vertebrate species and earlier developmental stages.^5^ Similar to bone marrow stromal cells (BMSCs, or perivascular SSCs) that maintain the adult skeleton,^6–9^ mouse and human growth plate SSCs generate chondrocytes, osteoblasts and hematopoietic stroma upon in vivo transplantation.^1,5^ However, they do not differentiate into adipocytes, highlighting the functional differences of SSCs from distinct developmental stages and skeletal compartments.^10,11^ Lineage-tracing studies in mice revealed multiple waves of osteoprogenitors during skeletal development.^12–14^ In contrast, the ontogeny of human embryonic SSCs during early skeletogenesis remains largely unknown. Given that the adult skeleton repairs in a way that recapitulates embryonic development, elucidation of the human embryonic SSC populations will definitely shed light on novel cell therapies that promote skeletal regeneration.

In vertebrates, the earliest progenitors of appendicular skeleton are formed within the limb buds.^15,16^ Limb patterning along the anterior–posterior (AP) axis is regulated by sonic hedgehog (SHH) signals from the zone of polarizing activity (ZPA),^17^ while the proximal–distal (PD) axis patterning is mainly regulated by FGF signals from the apical ectodermal ridge (AER).^18,19^ The distal mesenchymal cells underlying AER are undifferentiated and highly proliferative when receiving the FGF and WNT signals,^20,21^ which form the progress zone that elongates the limb buds. The core limb bud mesenchyme expresses *SOX9* to specify the osteo-chondrogenic lineage and generate the primordial cartilage template. Although different mesenchymal progenitors have been identified in mouse and chick limb buds,^22,23^ the cellular heterogeneity and lineage hierarchy within human limb buds remain unknown.

After chondrogenic differentiation of limb bud mesenchymal progenitors, long bones are generated by endochondral ossification.^24^ Blood vessels invade the center of the cartilage template with perichondrial osteoprogenitors to form the primary ossification center (POC),^12,14^ where osteoblasts, vascular endothelial cells, pericytes and hematopoietic cells populate to form the nascent bone marrow.^25–29^ In contrast, calvarial bones are generated by intramembranous ossification, which involves cranial mesenchyme condensation and direct mineralization on top of the cartilage anlagen.^30–33^ Whereas long bones are derived from lateral plate mesoderm, calvarial bones are derived from both neural crest and paraxial mesoderm that generate different parts of the calvarium.^34,35^ Interestingly, SSCs from mouse long bones and calvaria (or PSCs) share similar phenotypic markers (Lineage^–^CD51^+/low^Thy1^–^6C3^–^CD200^+^CD105^–^).^1,4^ Whether the embryonic long bones and calvaria contain skeletal stem/progenitor cells that share similar molecular features remains to be explored.

Single-cell RNA-sequencing (scRNA-seq) is a powerful tool in dissecting the cellular composition and lineage hierarchy within heterogeneous or rare cell populations.^36–38^ In the musculoskeletal system, a high-throughput scRNA-seq study during mouse embryonic development reported the transcriptional landscapes of AER, limb bud mesenchyme and skeletal muscle before POC formation.^39^ Recent scRNA-seq studies in adult mouse bone marrow also revealed the cellular heterogeneity of BMSCs, endothelial cells and osteo-chondrogenic lineage cells under homeostatic and stress conditions.^40–42^ scRNA-seq profiling during axolotl limb regeneration identified convergence of connective tissue cells back to multipotent skeletal progenitors that formed a limb bud-like blastema structure.^43^ In contrast, scRNA-seq studies of the human embryonic skeletogenesis are still lacking.

In this study, we generated the first comprehensive human embryonic skeletogenesis cell atlas by scRNA-seq. By systematically examining the cellular heterogeneity and lineage hierarchies within multiple skeletal compartments, we identified distinct skeletal stem/progenitor cells in human embryonic long bones and calvaria.

## Results

### Integrated analyses of single-cell transcriptomes during limb bud and long bone development

**Fig. 1:**
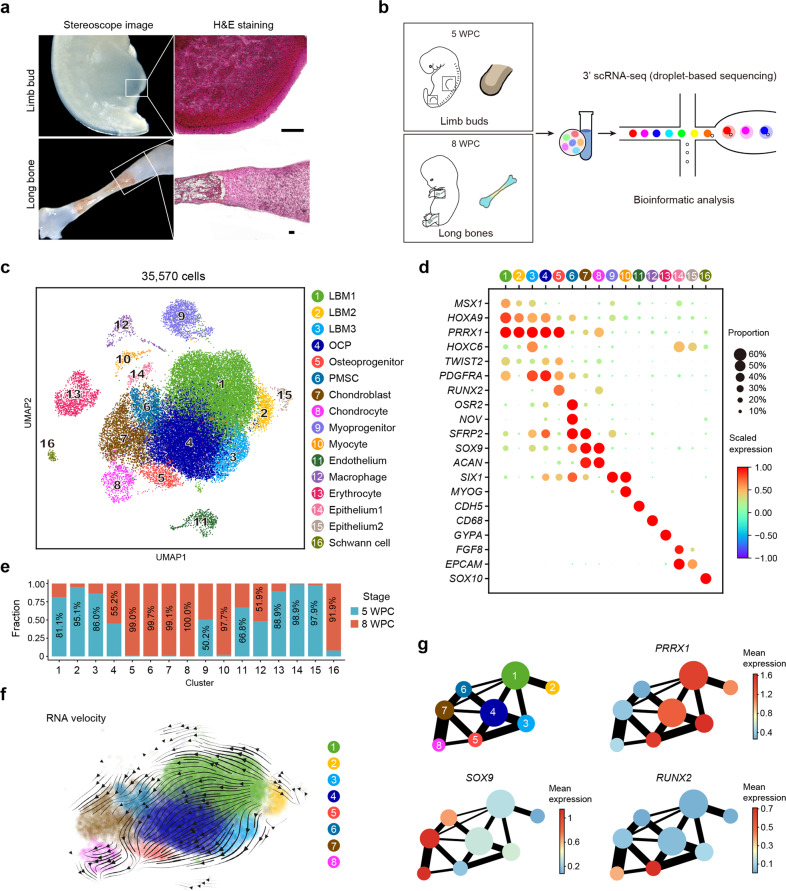
Integrated analysis of human limb buds and embryonic long bones. **a** Representative stereoscope images (left) and H&E images (right) of 5 WPC human limb bud and 8 WPC human long bone (*n* = 2 embryos). Scale bars, 100 μm. **b** Sampling workflow and experimental scheme. Human embryonic cells from 5 WPC limb buds and 8 WPC long bones were sorted and subjected to droplet-based scRNA-seq. **c** Distribution of 35,570 cells from limb buds and long bones. In total, 16 subsets were visualized by uniform manifold approximation and projection (UMAP). **d** Dot plots showing the expression of curated feature genes in 16 subsets. Dot size represented the proportion of cells expressing specific gene in the indicated subset and color bar represented the gene expression levels. **e** Proportion of cells from 5 WPC limb buds and 8 WPC long bones in each subset. **f** Developmental trajectory inferred by RNA velocity and visualized on the UMAP projection. **g** Partition-based graph abstraction (PAGA) showing the connectivity among subsets in **f**. The mean expression of representative genes (Mesenchymal, *PRRX1*; Chondrogenic, *SOX9*; Osteogenic, *RUNX2*) in each subset was shown in abstracted graph. Line thickness indicated the strength of connectivity. Color bar represents the gene expression levels.

To test whether SSCs exist during embryogenesis, we analyzed human limb buds at 5 weeks post conception (WPC), as well as human limb long bones at 8 WPC. Hematoxylin and eosin (H&E) staining showed condensed mesenchyme within limb buds, and the nascent bone marrow cavity in the center of long bones (Fig. 1a). To map the single-cell transcriptomes, upper and lower limb buds (5 WPC, *n* = 3 embryos from three independent experiments, Supplementary information, Fig. S1a), as well as forelimb and hindlimb long bones (8 WPC, *n* = 3 embryos from three independent experiments, Supplementary information, Fig. S1a) were dissected and subjected to enzymatic digestions. Dissociated cells were then sorted by flow cytometry to obtain live single cells for 3’ scRNA-seq on a 10× Genomics platform (Fig. 1b). After quality control and doublet exclusion, we obtained 19,890 single cells in 5 WPC limb buds and 15,680 single cells in 8 WPC long bones (Supplementary information, Fig. S1a). On average, we detected 2841 genes (10,212 unique molecular identities, UMI) per cell with less than 2.4% mitochondrial genes (Supplementary information, Fig. S1a). Normal karyotype was inferred by calculating copy number variation (CNV) scores on 100 randomly sampled cells for each embryo (Supplementary information, Fig. S1b).^44^ We performed canonical correlation analysis (CCA) to normalize variance and correct batch effects among different samples.^45^ Integrated analysis of the limb bud and long bone samples revealed 16 subsets (Fig. 1c; Supplementary information, Fig. S1c). The robustness of cell clustering was validated by calculating silhouette values (Supplementary information, Fig. S1d),^46^ and by random sampling and re-clustering analysis (Supplementary information, Fig. S1e).

We found three PRRX1^+^ limb bud mesenchymal subsets (LBM1–3) that mainly exist in 5 WPC limb buds (clusters 1–3), which differentially expressed *PDGFRA*, reflecting mesenchymal progenitors at different maturation stages (Fig. 1c–e).^22^ Notably, cluster 4 is a mesenchymal subset that equally distributed between limb bud and long bone samples, which expressed *PRRX1*, low level of *SOX9* and the highest level of *PDGFRA*, reminiscent of osteo-chondrogenic progenitors (OCPs) that give rise to long bones (Fig. 1c–e).^22^ EPCAM^+^ epithelial cells (clusters 14 and 15)^47^ and GYPA^+^ erythrocytes (cluster 13)^48^ were mainly detected in limb buds, while SIX1^+^ myoprogenitors (cluster 9),^49^ CDH5^+^ endothelial cells (cluster 11)^50^ and CD68^+^ macrophages (cluster 12)^51^ were found in both samples (Fig. 1c–e). In contrast, RUNX2^+^ osteoprogenitors (cluster 5),^52^ OSR2^+^NOV^+^ perichondrial mesenchymal stromal cells (PMSCs, cluster 6),^53,54^ SOX9^+^ chondroblasts and chondrocytes (clusters 7 and 8),^55^ MYOG^+^ myocytes (cluster 10),^56^ as well as SOX10^+^ Schwann cells (cluster 16)^57^ were mainly detected in long bones (Fig. 1c–e; Supplementary information, Table S1).

Pearson correlation analysis clearly distinguished the skeletogenic and non-skeletogenic subsets (Supplementary information, Fig. S1f). Pseudotime analysis by RNA velocity^58^ showed a differentiation continuum from limb bud mesenchymal progenitors to OCPs, followed by cell fate specification into osteogenic and chondrogenic lineages (Fig. 1f). Partition-based graph abstraction (PAGA) analysis^59^ showed a pivotal role of OCPs in linking limb bud mesenchymal progenitors (PRRX1^+^) to PMSC/chondroblasts/chondrocytes (SOX9^+^) and osteoprogenitors (RUNX2^+^) in embryonic long bones (Fig. 1g). Next, we separately analyzed the limb bud and long bone samples, with a special focus on the OCP lineage.

### Delineating mesenchymal lineage specification during limb bud development

**Fig. 2:**
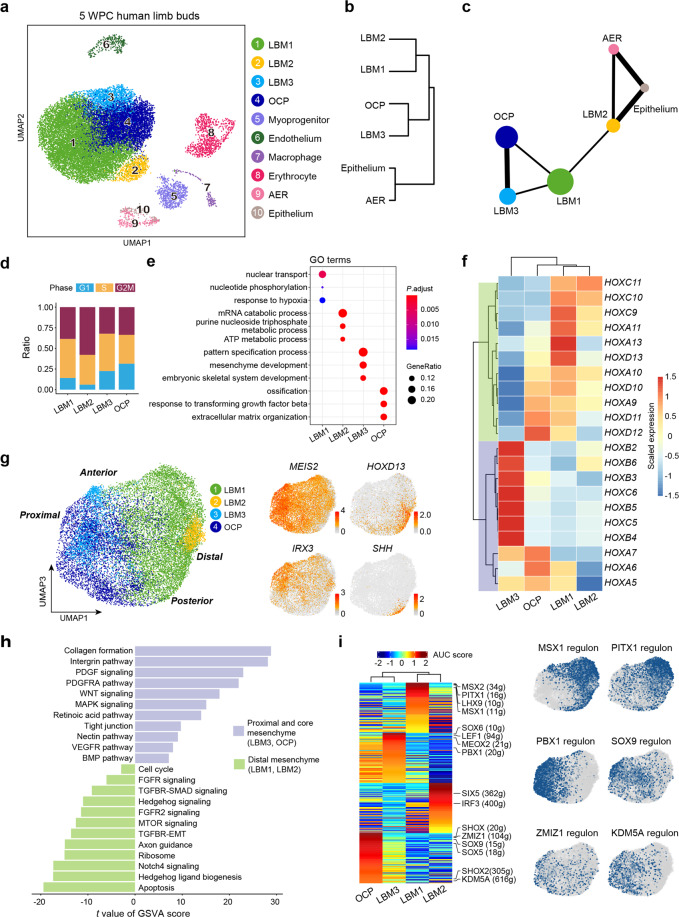
Characterization of human limb bud mesenchyme and epithelium. **a** UAMP visualization of the ten subsets in 5 WPC limb buds. **b** Hierarchical clustering of the mesenchymal and epithelial subsets using top 50 principal components (PCs). **c** The inferred relationships among the mesenchymal and epithelial subsets in PAGA layout. **d** Stacked bar charts showing the cell cycle distributions in the mesenchymal subsets. **e** Enriched GO terms of differentially expressed genes (DEGs) in the mesenchymal subsets. **f** Heatmap showing expression of curated *HOX* genes scaled across the mesenchymal subsets. *Hox* genes were clustered into two branches based on hierarchical clustering of the rows, as indicated in green and purple. **g** Visualization of the mesenchymal subsets (left) with UMAP plots showing the expression of curated PD and AP marker genes (right; Proximal, *MEIS2*; Distal, *HOXD13*; Anterior, *IRX3*; Posterior, *SHH*). **h** GSVA analysis of pathway enrichment in the proximal and core mesenchyme (LBM3/OCP) and distal mesenchyme (LBM1/2). *T* values for each pathway were shown (two-sided unpaired limma-moderated *t*-test). **i** Heatmap showing the area under the curve (AUC) score of regulons enriched in the mesenchymal subsets (left). *Z*-score (row scaling) was computed. Representative regulons were shown on the right. The number of predicted target genes for each regulon was shown in the parenthesis. Hierarchical clustering on columns indicated correlation between cell subsets. Binary activities of representative regulons were shown by UMAP plots (right).

We were able to identify 10 subsets in 5 WPC human limb buds (Fig. 2a). Hierarchical cluster analysis within the 4 mesenchymal subsets showed that LBM1 (cluster 1) clustered with LBM2 (cluster 2), while LBM3 (cluster 3) and OCP (cluster 4) clustered together (Fig. 2b). Of the two epithelial subsets (Fig. 2a), only cluster 9 (corresponds to Epithelium 1, Fig. 1c, d) highly expressed AER marker FGF8, consistent with previous study in mouse embryos.^39^ Surprisingly, PAGA analysis revealed a strong correlation between LBM2 and epithelial subsets (Fig. 2c), raising the possibility that LBM2 might represent progress zone mesenchyme that lies underneath the limb bud epithelium.^16,60^ Consistent with this hypothesis, cell cycle analysis showed that LBM2 was more proliferative as compared to other mesenchymal subsets, with more cells in G2/M phase (Fig. 2d). Gene ontology (GO) analysis showed that LBM2 was enriched with genes regulating metabolic processes, while LBM3 and OCP were enriched with genes involved in embryonic skeletal development and ossification (Fig. 2e).

During limb bud outgrowth, *HOX* gene expressions switch from 3′ to 5′ topologically associating domains along the PD axis.^61^ We found that LBM3 preferentially expressed 3′ *HOX* genes such as *HOX2*–*6*, while LBM1 and LBM2 preferentially expressed 5’ *HOX* genes such as *HOX9*–*11*, suggesting that they represented proximal (LBM3) and distal (LBM1 and LBM2) mesenchymal cells, respectively (Fig. 2f). In contrast, OCP expressed both 3’ and 5’ *HOX* genes, reminiscent of the core condensed mesenchyme that gives rise to the skeletal tissues (Fig. 2f). Consistent with this, when we aligned the mesenchymal subsets along the PD and AP axes using known marker genes such as *MEIS2*, *IRX3*, *HOXD13* and *SHH* (Fig. 2g), LBM3 and OCP were positioned at the proximal end, while LBM1 and LBM2 were positioned at the distal end (Fig. 2g). Of note, the distal most localization of LBM2 was in line with the position of progress zone. Consistent with previous studies,^62,63^ gene set variation analysis (GSVA) showed that the proximal and core mesenchyme was enriched with genes related to retinoic acid and PDGF signaling, while the distal mesenchyme was enriched with genes related to Hedgehog, FGF, TGFβ and Notch signaling (Fig. 2h).

To explore the gene regulatory networks (regulons) that determine cell fate specification in the mesenchymal subsets, we applied single-cell regulatory network inference and clustering (SCENIC) method to score the activity of regulons by an AUCell algorithm (AUC score), which reflects the co-expression of transcription factors (TFs) and their downstream target genes in each individual cell.^64^ Hierarchical clustering of the AUC scores again distinguished proximal/core and distal mesenchymal subsets (Fig. 2i). MSX1 and PITX1 regulons were enriched in LBM1 and LBM2,^65,66^ while PBX1 and SOX9 regulons were enriched in LBM3 and OCP.^22,67^ Interestingly, we also identified several OCP-specific regulons such as ZMIZ1 and KDM5A (Fig. 2i; Supplementary information, Table S2), which were shown to play important roles in neurodevelopmental disorder^68^ and osteoporosis,^69^ suggesting novel osteo-chondrogenic regulators within the limb bud mesenchyme.

To explore evolutionarily conserved and species-specific features during limb bud development, we analyzed a recently published scRNA-seq dataset of mouse hindlimb buds at similar embryonic stage (E11.5) (Supplementary information, Fig. S2a).^70^ SciBet is a recently developed algorithm that predicts cell identity by training multinomial model with given dataset.^71^ By training SciBet with our human dataset, we found that most human subsets were conserved in mouse except that LBM2 and epithelium (non-AER) subsets were not predicted in mouse limb buds (Supplementary information, Fig. S2b and Table S1). The lack of a highly proliferative LBM2 subset implied advanced maturation of E11.5 mouse limb buds (Supplementary information, Fig. S2a).^72^ Consistent with this, the mouse OCP subset highly expressed *SOX9* (Supplementary information, Fig. S2c), suggesting early chondrogenic differentiation. A much lower proportion of mouse AER was found within limb bud epithelium (6%) as compared to human AER (69%, Supplementary information, Fig. S2d), consistent with much shorter limbs in mouse.

Taken together, these data revealed the cellular heterogeneity and species-specific features of human limb bud mesenchyme and epithelium. Since osteogenesis is not initiated in 5 WPC human limb buds, we went on to analyze 8 WPC long bones in search of human embryonic SSCs.

### Delineating osteochondral lineage specification during long bone development

**Fig. 3:**
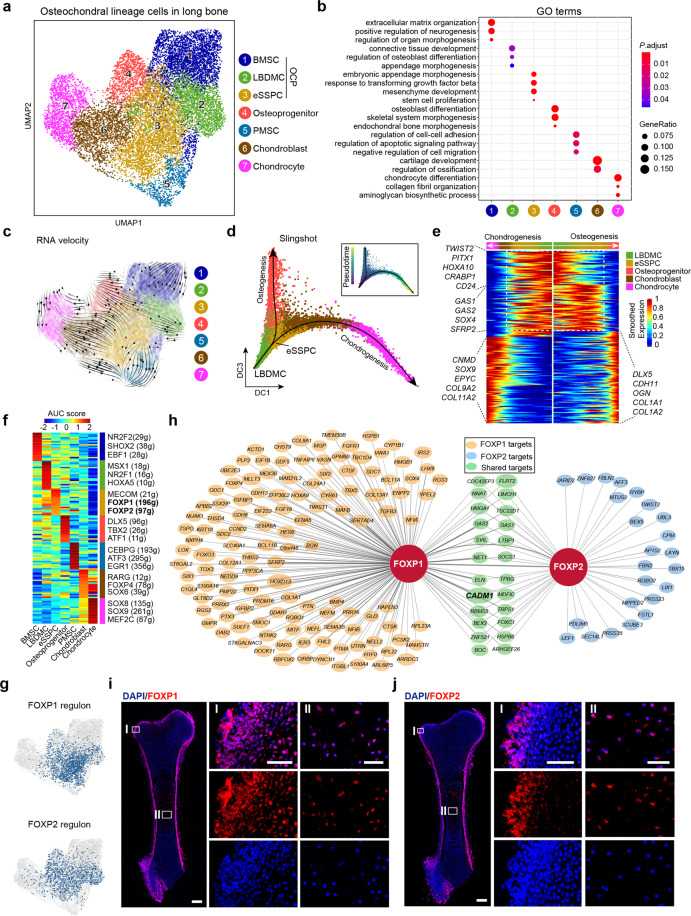
Characterization of the osteochondral lineage in human long bones identified embryonic SSCs. **a** UMAP visualization of seven OCLC subsets in 8 WPC human long bones. **b** Enriched GO terms of DEGs among the seven OCLC subsets. **c** Developmental trajectory of seven OCLC subsets inferred by RNA velocity and visualized on the UMAP projection. **d** Diffusion map visualization of the osteogenic and chondrogenic trajectories simulated by Slingshot across LBDMC, eSSPC, osteoprogenitor, chondroblast and chondrocyte subsets. The corresponding diffusion pseudotime was indicated in the upper right frame. **e** Heatmap of gene expressions (smoothed over 20 adjacent cells) in LBDMC, eSSPC, osteoprogenitor, chondroblast and chondrocyte subsets ordered by pseudotime of osteogenesis and chondrogenesis in **d**. Top 200 genes were selected according to the *P* values of GVM test and representative genes were shown. Shared genes in the two trajectories were indicated in dashed box. **f** Heatmap showing the AUC score of regulons enriched in human OCLC subsets. *Z*-score (row scaling) was calculated. Representative regulons were shown on the right. The number of predicted target genes for each regulon was shown in the parenthesis. **g** Binary activities of FOXP1 and FOXP2 regulons were shown by UMAP plots. **h** The FOXP1 and FOXP2 regulon networks in OCLC subsets. Line thickness indicated the level of GENIE3 weights. Dot size indicated the number of enriched TF motifs. **i**, **j** Immunofluorescent images of FOXP1 (**i**) and FOXP2 (**j**) expression in 8 WPC human femur. FOXP1/2^+^ cells were detected in the perichondrium (I) and inside POC (II). Merged and single-channel images of FOXP1/2 (red) and DAPI (blue) were shown (*n* = 2 embryos). Scale bars in snapshot images, 200 μm; scale bars in magnified images, 50 μm.

We analyzed the long bone dataset from 8 WPC human embryos (Supplementary information, Fig. S3a) and divided the osteochondral lineage cells (OCLCs) into 7 subsets (Fig. 3a). In addition to previously identified osteoprogenitor, PMSC, chondroblast and chondrocyte subsets (Fig. 3a, clusters 4–7), the OCP subset as revealed by integrated analysis (Fig. 1c) was subdivided into 3 subsets (clusters 1–3). Cluster 1 highly expressed *CXCL12* and *PDGFRA* (Supplementary information, Fig. S3b and Table S1), which are classical markers of BMSCs.^28,73^ Cluster 2 highly expressed limb bud mesenchymal genes such as *TWIST2* (Supplementary information, Fig. S3a, b and Table S1), which functions as an inhibitor of osteoblastic differentiation,^74^ reminiscent of limb bud-derived mesenchymal cells (LBDMCs). Cluster 3 highly expressed *GAS2* and localized in the center of all OCLC subsets (Fig. 3a; Supplementary information, Fig. S3a, b and Table S1). GO analysis showed significant enrichment of genes related to organ and appendage morphogenesis in clusters 1–3 (Fig. 3b). Interestingly, genes related to stem cell proliferation were enriched in cluster 3 (Fig. 3b), suggesting that it might contain embryonic skeletal stem/progenitor cells (eSSPCs).

Next, pseudotime analysis by RNA velocity was performed to explore the lineage relationships among OCLC subsets (Fig. 3c). We observed strong directional streams from eSSPC toward osteoprogenitor, chondroblast/chondrocyte and PMSC subsets (Fig. 3c). Interestingly, LBDMC was upstream of both eSSPC and BMSC, which formed two differentiation trajectories (Fig. 3c). Diffusion map analysis of LBDMC, eSSPC, chondroblast/chondrocyte and osteoprogenitor subsets simulated two differentiation trajectories featuring chondrogenesis and osteogenesis (Fig. 3d). Consistent with the RNA velocity analysis, eSSPC was located at the branching point of osteogenesis and chondrogenesis (Fig. 3d). When LBDMC was set as the root to identify temporally expressed genes over pseudotime, we found that genes highly expressed in LBDMCs (e.g., *PITX1*, *HOXA10*, *CRABP1*, *CD24*) and eSSPCs (e.g., *GAS1/2*, *SOX4* and *SFRP2*) were gradually down-regulated, while genes that highly expressed in chondrocytes (e.g., *CNMD*, *EPYC*, *COL9A2*, *COL11A2*) and osteoprogenitors (e.g., *DLX5*, *CDH11*, *OGN* and *COL1A1/2*) were up-regulated upon terminal differentiation (Fig. 3e). SCENIC analysis showed that eSSPCs were highly enriched with regulons such as FOXP1 and FOXP2 (Fig. 3f; Supplementary information, Table S2). The FOXP1 regulon seemed to be more specific to eSSPCs, as the FOXP2 regulon was also enriched in LBMDCs and osteoprogenitors (Fig. 3g). Nevertheless, FOXP1/2 did share a significant amount of target genes in eSSPCs (Fig. 3h).

Foxp1/2/4 are transcriptional repressors that are highly expressed in perichondral skeletal progenitors and proliferating chondrocytes during mouse endochondral ossification,^75^ which promote chondrogenesis and inhibit premature osteogenic differentiation.^75^ Consistent with this, immunofluorescent staining showed that FOXP1/2^+^ cells localized in the perichondrial regions of 8 WPC human long bones (Fig. 3i, j). A few FOXP1/2^+^ cells were also detected inside the POC, reminiscent of perichondrial skeletal progenitors that had invaded the cartilage template^12^ (Fig. 3i, j). We also analyzed a published scRNA-seq dataset of mouse hindlimb long bones at similar embryonic stage (E15.5) (Supplementary information, Fig. S3c, d).^70^ SciBet analysis found that human eSSPC was evolutionarily conserved in mouse long bones (Supplementary information, Fig. S3e). Interestingly, Foxp1/2/4 regulons were highly enriched in mouse eSSPCs (Supplementary information, Fig. S3f, g and Table S2), suggesting a fundamental role of FOXP family TFs in regulating eSSPCs during endochondral ossification.

Taken together, we identified three OCP subsets in 8 WPC human long bones, of which eSSPC represented a novel perichondrial subset that could potentially regulate long bone development and POC formation.

### Identification of CADM1 as a phenotypic marker of eSSPCs

**Fig. 4:**
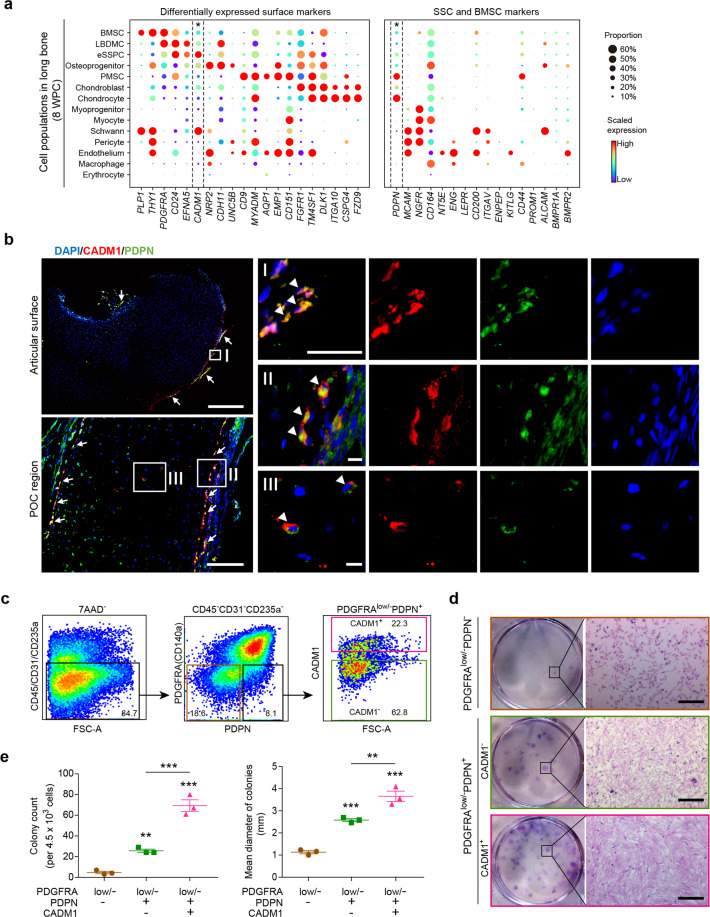
Identification of CADM1 as a phenotypic marker of eSSPCs. **a** Dot plots showing the expression of differentially expressed cell surface genes (left) and candidate SSC markers (right) in 8 WPC human long bone subsets. Asterisks indicated positive markers that were used to enrich eSSPCs. **b** Immunofluorescent images of PDPN^+^CADM1^+^ cells in 8 WPC human long bones. Overviews of PDPN^+^CADM1^+^ cells (arrows) in the articular (upper left) and POC (bottom left) regions were shown on the left. PDPN^+^CADM1^+^ cells were found in the inner layer of perichondrium in the articular regions (I) and surrounding POC (II). A few PDPN^+^CADM1^+^ cells were also found inside POC (III). Arrow heads indicated enlarged PDPN^+^CADM1^+^ cells. Merged and single-channel images of DAPI (blue), CADM1 (red) and PDPN (green) were shown (*n* = 2 embryos). Scale bars in snapshot images, 50 μm; scale bars in magnified images, 5 μm. **c** Flow cytometry gating strategies for sorting different populations in 8 WPC long bones (*n* = 3 embryos). **d** Representative crystal violet staining of CFU-F colonies generated by the sorted populations as indicated in **c**. Magnified images of the boxed areas were shown on the right. Scale bars, 25 μm. **e** Quantifications of the number (left) and mean diameter (right) of the CFU-F colonies (*n* = 3 embryos). The statistical significance of differences was determined using one-way ANOVA with multiple comparison tests (LSD). *P < 0.05; **P < 0.01; ***P < 0.001. Error bars indicated SEM.

To prospectively isolate eSSPCs for functional characterizations ex vivo, we first screened for cell surface markers that were differentially expressed among long bone OCLC subsets. Interestingly, we found that the cell adhesion molecule *CADM1* was preferentially expressed in eSSPCs (Fig. 4a). SCENIC analysis showed that FOXP1/2-binding motifs were highly enriched in the predicted *cis*-regulatory elements of *CADM1* among all co-expressed target genes (Fig. 3h), suggesting that it could be used as a legitimate phenotypic marker of eSSPCs. Since *CADM1* was also expressed in Schwann cells (Fig. 4a), we sought to further enrich eSSPCs by combining with previously reported SSC or BMSC markers (Fig. 4a).^1,5,11^ Interestingly, we found that *PDPN* was differentially expressed in eSSPCs (PDPN^+^) and Schwann cells (PDPN^–^) (Fig. 4a). Immunofluorescent staining of CADM1 and PDPN on 8 WPC human long bone sections showed that PDPN^+^CADM1^+^ cells mainly localize in the perichondrial regions and inside the POC (Fig. 4b; Supplementary information, Fig. S4b), which were reminiscent of the FOXP1/2^+^ cells (Fig. 3i, j).

In silico transcript-averaged cell scoring (TACS) analysis^76^ revealed that the purity of eSSPCs could be further improved by enrichment of PDGFRA^low/–^PDPN^+^CADM1^+^ cells among OCLC subsets (Supplementary information, Fig. S4a). In contrast, *THY1* (CD90), *NGFR* (CD271), *MCAM* (CD146) or *NT5E* (CD73) were hardly detected in eSSPCs (Fig. 4a; Supplementary information, Fig. S4a). Next, we sorted PDGFRA^low/–^PDPN^–^, PDGFRA^low/–^PDPN^+^CADM1^–^ and PDGFRA^low/–^PDPN^+^CADM1^+^ cells from 8 WPC human long bones by flow cytometry (Fig. 4c), and performed colony-forming unit-fibroblast (CFU-F) and mesenchymal sphere cultures to assess their colony- and sphere-forming efficiencies ex vivo. As compared to PDGFRA^low/–^PDPN^–^ cells (4.67 ± 1.20 colonies per 4.5 × 10^3^ cells and 1.13 ± 0.07 mm diameters), PDGFRA^low/–^PDPN^+^CADM1^–^ cells showed significantly higher colony-forming efficiency with colonies of larger size (25.67 ± 1.67 colonies per 4.5 × 10^3^ cells and 2.58 ± 0.06 mm diameters, Fig. 4d, e). Remarkably, PDGFRA^low/–^PDPN^+^CADM1^+^ cells showed an even higher colony-forming efficiency with significantly more colonies of larger size (69.33 ± 5.61 colonies per 4.5 × 10^3^ cells and 3.66 ± 0.23 mm diameters) as compared to PDGFRA^low/–^PDPN^+^CADM1^–^ cells (Fig. 4d, e). Mesenchymal sphere formation analyses showed similar results (Supplementary information, Fig. S4c, d). PDGFRA^+^ cells showed lower colony-forming efficiency with colonies of smaller size (13.00 ± 1.00 colonies per 4.5 × 10^3^ cells and 2.68 ± 0.21 mm diameters) as compared to PDGFRA^low/–^PDPN^+^CADM1^+^ cells (Supplementary information, Fig. S4e, f) and exhibited trilineage differentiation potential (Supplementary information, Fig. S4g, h), reminiscent of BMSCs in the nascent POC.

To further validate that these phenotypic markers indeed enrich eSSPCs, we performed scRNA-seq analysis on flow cytometrically sorted PDGFRA^low/–^PDPN^+^CADM1^+^ cells. Clustering analysis revealed two distinct subsets, including 93.3% eSSPCs that expressed *FOXP1*, *FOXP2* and *GAS2*, and 6.7% Schwann cells that expressed *SOX2*, *SOX10* and *MPZ* (Supplementary information, Fig. S5a, b). Similarity analysis also confirmed that eSSPCs were highly enriched in PDGFRA^low/–^PDPN^+^CADM1^+^ cells (Supplementary information, Fig. S5c).

### eSSPCs self-renew and undergo osteo-chondrogenic differentiation

**Fig. 5:**
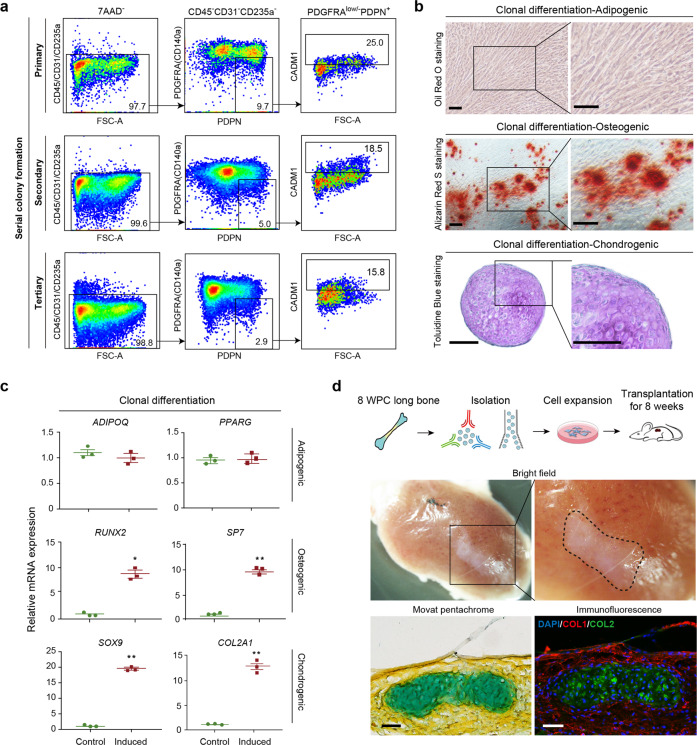
Functional characterizations of eSSPCs in vitro and in vivo. **a** Flow cytometry plots showing the maintenance of phenotypic eSSPCs after serially passaging clonally expanded PDGFRA^low/–^PDPN^+^CADM1^+^ cells (*n* = 3 clones). **b** Representative oil red O (top), alizarin red (middle) and toluidine blue (bottom) staining after adipogenic, osteogenic and chondrogenic differentiation of clonally expanded eSSPCs (PDGFRA^low/–^PDPN^+^CADM1^+^). Magnified images of the boxed areas were shown on the right. Scale bars, 200 μm. **c** qPCR analyses of adipogenic, osteogenic and chondrogenic marker genes in clonally expanded eSSPCs before and after trilineage differentiation in vitro (*n* = 3 clones). The statistical significance of differences was determined using Wilcoxon signed rank test. *P < 0.05; **P < 0.01. Error bars indicated SEM. **d** Renal subcapsular transplantation. The work flow for functional characterization of eSSPC in vivo (top). Subcapsular xenografts were dissected and sectioned 8 weeks after transplantation of culture expanded eSSPCs into immunodeficient mice. Bright field (middle), Movat pentachrome staining (bottom left; cartilage, blue; bone and fibrous tissue, yellow) and immunofluorescent staining images (bottom right; DAPI, blue; collagen I (COL1), red; collagen II (COL2), green) were shown (*n* = 9 grafts from three embryos). Scale bars, 50 μm.

To test the self-renewal and differentiation potentials of eSSPCs, we first sorted PDGFRA^low/–^PDPN^+^CADM1^+^ cells to perform serial CFU-F colony formation assay. Single CFU-F colonies formed by flow cytometrically sorted PDGFRA^low/–^PDPN^+^CADM1^+^ cells were clonally expanded and serially passaged, which could generate secondary and tertiary colonies that maintained the eSSPC immunophenotypes (Fig. 5a; Supplementary information, Fig. S5d). Next, we performed in vitro trilineage differentiation on nonclonal and clonal cultures (cells were clonally expanded from single CFU-F colonies) of PDGFRA^low/–^PDPN^+^CADM1^+^ cells, and found that they underwent osteogenic and chondrogenic differentiation, but not adipogenic differentiation (Fig. 5b; Supplementary information, Fig. S5e). In contrast, PDGFRA^low/–^PDPN^+^CADM1^–^ cells only underwent osteogenic differentiation (Supplementary information, Fig. S5g). The differentiation efficiencies were quantified by quantitative real-time PCR (qPCR) analysis of adipogenic (*ADIPOQ* and *PPARG*), osteogenic (*RUNX2* and *SP7*) and chondrogenic (*SOX9* and *COL2A1*) marker genes (Fig. 5c; Supplementary information, Fig. S5f, h).

To test the in vivo differentiation potentials of PDGFRA^low/–^PDPN^+^CADM1^+^ and PDGFRA^low/–^PDPN^+^CADM1^–^ cells, we performed renal subcapsular transplantation of nonclonal cell cultures in immunodeficient mice. Eight weeks after transplantation, the subcapsular grafts were harvested and sectioned. Movat pentachrome staining and immunofluorescent staining of collagen I and II revealed osteo-chondrogenic differentiation of PDGFRA^low/–^PDPN^+^CADM1^+^ (Fig. 5d). In contrast, PDGFRA^low/–^PDPN^+^CADM1^–^ cells underwent osteogenic but not chondrogenic differentiation (Supplementary information, Fig. S5i). We did not observe bone marrow formation in subcapsular grafts generated by PDGFRA^low/–^PDPN^+^CADM1^+^ or PDGFRA^low/–^PDPN^+^CADM1^–^ cells, suggesting that they are functionally distinct from growth plate human SSCs that could organize a hematopoietic microenvironment.^5^ Taken together, these data suggested that CADM1 is an important phenotypic marker of eSSPCs, and that PDGFRA^low/–^PDPN^+^CADM1^+^ cells enriched self-renewing eSSPCs that generate the osteochondral lineages during long bone development, but fail to reconstitute the bone marrow microenvironment.

### Delineating osteogenic lineage specification during calvaria development

**Fig. 6:**
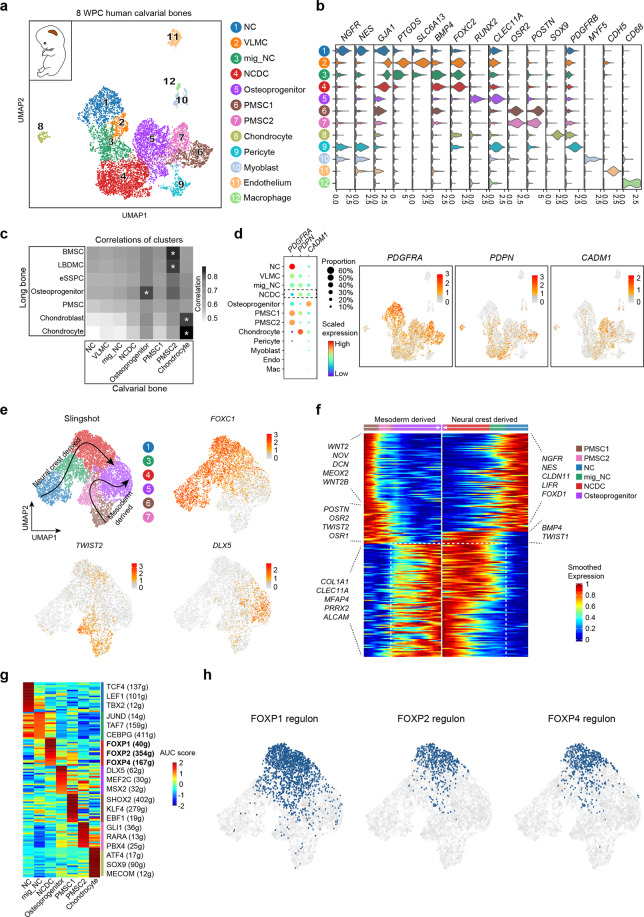
Characterization of the osteogenic lineages in human embryonic calvaria identified neural crest-derived skeletal progenitors. **a** UMAP visualization of 12 subsets in 8 WPC calvarial bones (*n* = 2 embryos). Inset illustrated the position of calvarial bone. **b** Violin plots showing the expression of feature genes for each subset. **c** Heatmap showing the transcriptome correlation between osteogenic subsets in calvarial and OCLC subsets in long bone. Asterisks indicated subsets with correlation coefficients > 0.8. **d** Dot plots (left) and UMAP plots (right) showing the expression of eSSPC marker genes in 12 subsets of 8 WPC calvarial. **e** UMAP visualization of the two osteogenic trajectories simulated by Slingshot across NC, mig_NC, NCDC, osteoprogenitor, PMSC1 and PMSC2 subsets (Upper left). Expression UMAP plots of marker genes (NC, *FOXC1*; Mesoderm, *TWIST2*; Osteoprogenitor, *DLX5*). **f** Heatmap of the gene expressions (smoothed over 20 adjacent cells) in subsets ordered by pseudotime of osteogenesis as in **e**. Top 200 genes were selected according to the *P* values of GVM test and representative genes were shown. Shared genes in two trajectories were indicated in dashed box. **g** Heatmap showing the AUC scores of regulons enriched in the osteogenic subsets. *Z*-score (row scaling) was computed. Representative regulons were shown on the right. **h** Binary activities of FOXP1/2/4 regulons were shown by UMAP plots.

To test whether similar skeletal stem/progenitor cells exist in the embryonic calvarium, we performed scRNA-seq in 8 WPC human calvaria (*n* = 2 embryos from two independent experiments, Supplementary information, Fig. S6a). Analysis of 7287 CD235a^–^7AAD^–^ (live non-erythrocytes) single cells revealed 12 distinct subsets (Fig. 6a), which included: (1) NGFR^+^ cranial neural crest (NC) cells (cluster 1) that highly expressed *NES*^77^; (2) two GJA1^+^ subsets including vascular leptomeningeal cells (cluster 2, VLMCs) that highly expressed *SLC6A13* and *PTGDS*,^78^ and migratory NC (mig_NC) cells that expressed higher level of *BMP4* (cluster 3)^79,80^; (3) neural crest-derived cells (cluster 4, NCDC) that highly expressed *BMP4* and *FOXC2*^81^; (4) RUNX2^+^ osteoprogenitors (cluster 5) that highly expressed osteogenic factors *DLX5* and *CLEC11A*^82,83^; (5) two OSR2^+^ PMSC subsets (clusters 6 and 7) that highly expressed *POSTN*; (6) SOX9^+^ chondrocytes that highly expressed *COL9A2* (cluster 8); (7) PDGFRB^+^ pericytes that highly expressed *MCAM* and *ACTA2*; (8) MYF5^+^ myoblasts; (9) CDH5^+^ endothelial cells and (10) CD68^+^ macrophages (Fig. 6a, b; Supplementary information, Table S1). We did not identify Schwann cell population in 8 WPC human calvaria. The only calvarial subset that expressed low levels of *SOX10*, *NES*, *PLP1* and *ERBB3* was the myoblast population (data not shown).

As compared to 8 WPC long bones, higher proportions of osteoprogenitors and PMSCs but much lower proportion of chondrocytes were detected in 8 WPC calvarial bones (Supplementary information, Fig. S6b), highlighting the fundamental differences between endochondral and intramembranous ossification.^15^ Spearman correlation analysis showed that calvarial chondrocyte and osteoprogenitor subsets were more correlated with their long bone counterparts (Fig. 6c), while the PMSC2 subset seemed to be closely related to LBDMC and BMSC subsets in long bones (Fig. 6c). Integrated analysis of all subsets at the pseudo-bulk level showed similar results (Supplementary information, Fig. S6c). Although no calvarial subset highly resembled long bone eSSPC at the transcriptome level, we found that NCDC shared similar phenotypic markers to long bone eSSPC (PDGFRA^low/–^PDPN^+^CADM1^+^) (Fig. 6d), which located in between mig_NC cells and osteoprogenitors (Fig. 6a). Immunostaining of 8 WPC human calvarial sections showed that PDPN^+^CADM1^+^ cells localized in the outer layer of sagittal suture (Supplementary information, Fig. S6d), reminiscent of PSCs in the adult mouse calvarium.^4^

Next, we performed pseudotime analysis within osteogenic subsets by Slingshot,^84^ which revealed two distinct differentiation trajectories (Fig. 6e). Specifically, the FOXC1^+^ NC lineage cells and TWIST2^+^ mesodermal lineage cells converge to generate DLX5^+^ osteoprogenitors (Fig. 6e), where NCDC seemed to play a pivotal role in the transition from mig_NC cells to osteoprogenitors (Fig. 6e). Gene expression analysis showed that NC lineage cells down-regulated neural genes such as *NGFR, NES* and *CLDN11*^85^ to generate NCDCs and osteoprogenitors (Fig. 6f). In contrast, mesodermal lineage cells down-regulated WNT signaling genes such as *WNT2* and *WNT2B*, as well as TFs like *MEOX2*, *OSR1* and *OSR2* to generate osteoprogenitors (Fig. 6f). Calvarial osteoprogenitors highly expressed *COL1A1*, *PRRX2* and *CLEC11A*, a recently identified osteogenic factor that promotes the maintenance of adult skeleton.^83,86^ GSVA analysis showed that EPH-EPHRIN, WNT-LPR6 and RAC1 activation pathways were enriched in NCDCs (Supplementary information, Fig. S6e). Similar to long bone eSSPC, SCENIC analysis showed that FOXP1/2 regulons were highly enriched in NCDC (Fig. 6g, h; Supplementary information, Fig. S6f and Table S2), although few FOXP1/2 target genes were shared by these two subsets (Fig. 3h; Supplementary information, Fig. S6f). Interestingly, the FOXP4 regulon was also enriched in NCDC (Fig. 6g, h), which formed an integrated transcriptional network with FOXP1/2 (Supplementary information, Fig. S6f), suggesting critical roles of FOXP family TFs in NCDC specification. Taken together, these data revealed two distinct routes of osteogenic differentiation in calvaria, and identified NCDC as a potential skeletal stem/progenitor cell subset mediating intramembranous ossification during human calvarial development.

## Discussion

Whereas skeletogenesis has been extensively studied in model organisms such as mouse, chick and axolotl,^39,43,70,87^ human studies largely remain at the histomorphological level. In 2018, Ferguson et al. interrogated 17 WPC human fetal musculoskeletal subsets by bulk RNA-seq and compared chondrocyte features among 4 developmental stages.^88^ Recently, a human skeletal muscle atlas was reported during embryonic, fetal and postnatal development.^89^ Here, we provide the first transcriptional landscape of human embryonic skeletogenesis at single-cell resolution and shed light on novel skeletal stem/progenitor cells orchestrating lineage specifications during endochondral and intramembranous ossification. Together with the previous studies, we are now approaching a better understanding of the ontogeny of human musculoskeletal system.

Although the patterning mechanisms during limb bud development have been well-studied and simulated by different models,^15,17,19^ the heterogeneity of human limb buds has been elusive. We identified 4 mesenchymal and 2 epithelial subsets in 5 WPC human limb buds. By analyzing *Hox* gene expression and well-known marker genes, we were able to align the 4 mesenchymal subsets along PD and AP axes (Fig. 2f, g). Importantly, we identified a highly proliferative LBM2 subset at the distal most mesenchyme, implicating immature mesenchymal progenitors underlying AER.^21,65^ We also identified an OCP subset with chondrogenic potential in the core mesenchyme. Unlike mouse limb bud mesenchymal progenitors (Sox9^–^Pdgfra^hi^) and OCPs (Sox9^+^Pdgfra^hi^),^22^ human limb bud OCPs are SOX9^low^PDGFRA^hi^ (Fig. 1d), suggesting that they are less differentiated than mouse OCPs. Interestingly, the E11.5 mouse limb buds lacked an equivalent LBM2 subset, showed early chondrogenic differentiation of OCP, and contained fewer proportion of AER cells (Supplementary information, Fig. S2). Together, these data suggested greater potential of human limb bud outgrowth that could possibly contribute to longer limb bones. Whether the novel regulons identified in human limb bud OCP (e.g., ZMIZ1 and KDM5A) critically control osteo-chondrogenic differentiation remains to be validated by functional studies.

We identified 3 subsets of skeletal stem/progenitor cells in 8 WPC human long bones, namely, LBDMC, BMSC and eSSPC. The LBDMC expressed marker genes of limb bud mesenchymal progenitors, and was inferred by pseudotime analysis to generate BMSCs and eSSPCs (Fig. 3c). This suggested that it might be derived from the peripheral limb bud mesenchyme surrounding the condensed cartilage template. Whether LBDMC could function as embryonic SSCs that could generate the entire limb bones or give rise to growth plate SSCs in the fetal stage remain to be explored by future studies. The BMSC subset highly expressed *PDGFRA* and *CXCL12*, but not *LEPR* or *ADIPOQ* (data not shown), suggesting their differences with adult mouse BMSCs.^6,41,90^ Similar to perivascular SSCs in the adult human bone marrow,^26^ PDGFRA^+^ cells from 8 WPC human long bones underwent trilineage differentiation in vitro (Supplementary information, Fig. S4e–h), which were in sharp contrast to eSSPCs that only showed osteo-chondrogenic differentiation potential (Fig. 5b, c). Considering that eSSPCs mainly localized in the perichondrial regions of embryonic long bones, these data indicated that BMSC and eSSPC represent stem/progenitor subsets from different skeletal compartments, which is consistent with the current notion that skeletal development and repair is maintained by multiple populations of SSCs.^10^

An equivalent eSSPC subset was also found in E15.5 mouse embryonic long bones (Supplementary information, Fig. S3), suggesting that it is an evolutionary conserved population. The fact that both human and mouse eSSPCs were enriched with FOXP1/2 regulons was quite intriguing (Fig. 3i), since mouse Foxp1/2/4 are highly expressed in perichondrial skeletal progenitors.^75^ Foxp1/2/4 regulate endochondral ossification by interacting with Runx2 to repress its transactivation activity,^75^ which partially explain how eSSPCs are maintained in an undifferentiated state. In contrast to FOXP1/2, the FOXP4 regulon activity was enriched in the chondroblast subset (Fig. 3f), suggesting that it is important for chondrogenesis. Notably, much more FOXP2 target genes were found in human long bones as compared to mouse (human: 97, mouse: 12), consistent with the recent discovery that skeletal FOXP2 contributes to the acquisition of important human traits such as language and bipedalism.^91^ More functional studies are needed to fully address whether FOXP1/2 regulate human eSSPC self-renewal and differentiation, as well as the underlying molecular mechanisms.

CADM1 was previously identified as an osteoblastic adhesion molecule and a diagnostic marker for osteosarcoma.^92^ Here we found that CADM1 enriched eSSPCs in the perichondrium of 8 WPC human long bones. Similar to human growth plate SSCs,^5^ eSSPCs exhibit high clonogenic capacity, which self-renew and undergo osteo-chondrogenic but not adipogenic differentiation in vitro and in vivo. However, eSSPCs do not form bone marrow upon renal subcapsular transplantation, probably due to the fact that they mainly localized in the perichondrium. In contrast to eSSPCs, PDGFRA^low/–^PDPN^+^CADM1^–^ cells only underwent osteogenic differentiation in vitro and in vivo, suggesting that they might function as progenitor cells mediating intramembranous ossification in the perichondrium.^93^ We were unable to transplant uncultured eSSPCs due to limited number of cells we could obtain from 8 WPC human long bones. Future optimization of the transplantation protocol is needed to further dissect the in vivo functions of human and mouse eSSPCs. Furthermore, genetic lineage-tracing studies would help elucidate the relationships between eSSPCs and postnatal SSCs from the growth plate, periosteum and perivascular regions in mouse models.

Interestingly, we also identified a NCDC subset in 8 WPC human calvaria that shared similar phenotypic markers with long bone eSSPC (Fig. 6d). Importantly, FOXP1/2/4 regulons were all enriched in calvarial NCDCs, suggesting fundamental role of FOXP family TFs in regulating intramembranous ossification. Consistent with this, *Foxp1/2* were detected in skeletal progenitors during mouse craniofacial bone development,^94^ while deficiencies of Foxp1/2/4 led to craniofacial defects.^75^ Unlike long bone eSSPCs, NCDCs are clearly separated from chondrocytes at both single-cell (Fig. 6a) and pseudo-bulk levels (Supplementary information, Fig. S6c), which was characteristic of intramembranous ossification. Future studies are needed to test whether NCDCs are evolutionarily conserved in mouse embryonic calvarium, and to prospectively isolate NCDCs for functional analysis of their stem cell activities. Furthermore, the relationships between embryonic NCDCs and calvarial PSCs in postnatal mice could be addressed by genetic lineage-tracing studies.^4^

Taken together, the human skeletal stem/progenitor cells and skeletogenic mechanisms we uncovered here might help develop novel cell therapies to promote bone and cartilage regeneration, which could treat skeletal disorders such as non-union fracture, osteoporosis and craniofacial defects.

## Materials and methods

### Human embryonic sample collection

Healthy human embryonic samples were obtained with elective medical termination of pregnancy in the Academy of Military Medical Sciences (the Fifth Medical Center of the PLA General Hospital). All human studies were conducted in accordance with the official ethical guidelines and protocols approved by the Ethics Committee of the Affiliated Hospital of Academy of Military Medical Sciences (ky-2017-3-5). The written informed consents were obtained from donors before embryo collection by elective medical termination of pregnancy. Donors with chronic, infectious, genetic diseases or abnormal pregnancy were excluded in this study. Morphological examinations were performed carefully under the stereoscope for each embryo, and CNVs were evaluated using scRNA-seq data to confirm normal karyotypes (see details below). Days post fertilization of embryos were determined according to the measurement of crown-rump length (CRL) and number of somite pairs, and staged into 5 and 8 WPC.^95^ The gender of embryos used for scRNA-seq was identified based on the expression of XIST (female) and RPS4Y1 (male).^96^ Sample information was summarized in Supplementary information, Figs. S1a and S6a. The morphology of the embryonic limb bud and long bone was assessed by Hematoxylin-Eosin Staining Kit (Fig. 1a).

### Mice

NOG (NOD.Cg-Prkdc^scid^Il2rg^tm1Sug^/JicCrl) immunodeficient mice (Beijing Vital River Laboratory Animal Technology Co., Ltd.) were used as recipients for renal subcapsular transplantation of human eSSPCs. All procedures and protocols were approved by the Ethics Committee of the Academy of Military Medical Sciences (the Fifth Medical Center of the PLA General Hospital).

### Preparation of single-cell suspensions from human limb buds and long bones

Human embryonic limb buds were isolated and transferred to IMDM medium (Gibco) containing 10% fetal bovine serum (FBS) (HyClone) on ice. The tissues were washed with phosphate-buffered saline (PBS) and transferred to pre-warmed digestion medium containing 0.1 g/mL Collagenase I (Sigma) and 0.1 g/mL Collagenase II (Sigma). After vigorous shaking, the samples were incubated at 37 °C for 30 min with gentle shaking every 5 min. Digestion was terminated by adding IMDM medium containing 10% FBS. After centrifugation at 350× *g* for 6 min, collected cells were resuspended in FACS sorting buffer (1× PBS with 1% BSA) for subsequent staining. For long bone specimens, forelimbs and hindlimbs were dissected to obtain humeri, ulnae, radii, femurs, tibiae and fibulae after removing skeletal muscles and connective tissues. For calvarial bone specimens, frontal bones, parietal bones and occipital bones were dissected. All procedures were performed on ice. Whole bones, including the perichondrium/periosteum and bone marrow, were cut into small pieces by scissors and then subjected to enzymatic digestion as described above. The digested tissues were then ground by a syringe plunger and filtrated with 40 μm strainer to remove cartilage or bone chips, after which cells were centrifugated and resuspended in FACS sorting buffer. The viability of cells was 80%–90% by trypan blue staining (0.4%) and 70%–80% by 7-AAD staining.

### Flow cytometry

The following antibodies were used: CD45-APC-Cy7 (BD, 557833, 1:50), CD31-Biotin (eBioscience, 13-0319-82, 1:50), Steptavidin-APC-eFlour780 (eBioscience, 47-4317-82, 1:100), CD235a-APC-Cy7 (Biolegend, 349116, 1:50), CD140a-BB515 (BD, 564594, 1:50), PDPN-APC (eBioscience, 17-9381-41, 1:50) and CADM1-PE (MBL, CM004-5, 1:50). Cells were stained in sorting buffer (PBS + 1% BSA) for 30 min at 4 °C, washed once and resuspended in sorting buffer with 7-AAD (eBioscience, 00-6993-50, 1:50) as live cell dye. Flow cytometry was performed on BD FACS Aria II. Pre-gating was first done for live cells based on 7-AAD staining. Gating strategies were based on Fluorescence Minus One (FMO) controls. FlowJo v10 software was used for analyzing the flow cytometry data.

### CFU-F culture and mesenchymal sphere assay

For CFU-F cultures, sorted cells were seeded in 6-well plate (4.5 × 10^3^ cells/well) containing culture medium (α-MEM supplemented with 10% FBS, 1% Penicillin/Streptomycin solution and 1 ng/mL bFGF) and incubated at 37 °C with 5% CO_2_. Half of the medium was changed every 3–4 days. At day 10, cells were fixed and stained with crystal violet staining solution. Adherent colonies with more than 50 cells were quantified. Serial CFU-F colony formation was performed by seeding sorted cells in culture medium at clonal density, and serially passaged to generate the secondary and tertiary colonies. For mesenchymal sphere assay, 4.5 × 10^3^ sorted cells were plated in a 6-well ultra-low adherent dish with culture medium and left undisturbed for a week.^73^ Half of the medium was changed every week, and the spheres were quantified at day 10.

### Adipogenic, osteogenic and chondrogenic differentiation assays

For nonclonal adipogenic and osteogenic differentiation, sorted cells were cultured for 10 days and replated at a density of 2.0 × 10^4^/cm^2^. Adipogenic differentiation was performed in DMEM (Gibco) supplemented with 10% FBS, 1% Penicillin/Streptomycin, 0.5 μM isobutylmethylxanthine (Sigma), 60 μM indomethacin (Sigma, 17378), 5 μg/mL insulin (Sigma) and 1 μM dexamethasone (Sigma, D2915) for 1 week (medium was changed every 3 days), and quantified by oil red O staining (Sigma) and qPCR. Osteogenic differentiation was performed in osteogenic medium (Cyagen, GUXMX-90021) for 3 weeks (medium was changed every 3 days) and quantified by alizarin red S staining (Sigma) and qPCR. The osteogenic differentiation medium contained α-MEM supplemented with 10% FBS, 1% Penicillin/Streptomycin, 1% glutamine, 50 μg/mL L-ascorbate acid, 10 mM β-glycerophosphate and 100 nM dexamethasone. For nonclonal chondrogenic differentiation, 2.5 × 10^5^ cultured cells were centrifugated at 1100 rpm in 15 mL polypropylene conical tubes to form pellets and cultured in chondrogenic medium for 3–4 weeks (medium was changed every 3 days). The chondrogenic medium contained high glucose DMEM (Corning) supplemented with 10 ng/mL TGFβ3 (Peprotech), 100 nM dexamethasone (Sigma), 50 μg/mL ascorbic acid-2-phosphate (Sigma), 1 mM sodium pyruvate (Gibco), 40 μg/mL proline (Sigma) and 1× ITS cell culture supplement (Cyagen) containing 6.25 μg/mL bovine insulin, 6.25 μg/mL transferrin, 6.25 μg/mL selenous acid, 5.33 μg/mL linoleic acid and 1.25 mg/mL BSA. Chondrogenic differentiation was quantified by cryosection of the cell pellets followed by toluidine blue staining and qPCR. For clonal trilineage differentiation, single cells were flow cytometrically sorted into 96-well plates to form single CFU-F colonies. Clonally expanded cells were split into three parts and allowed to differentiate in osteogenic, adipogenic and chondrogenic mediums as described above. Clonal chondrogenic differentiation was also validated by alcian blue and safranin O staining.

### RNA extraction and qPCR

Total RNA was extracted from cells using Trizol reagent (Invitrogen) according to the manufacturer’s instructions. cDNA was prepared using Transgene Reverse Transcription Kit (Transgene). qPCR reactions were prepared using SYBR Green Master Mix (Applied Biosystem) and run on a 7500 Real-Time PCR Systems (Applied Biosystems). A list of the primers used was provided in Supplementary information, Table S5. Human *GADPH* was used as loading control and the relative mRNA abundance was calculated using a comparative CT method.

### Renal subcapsular transplantation

PDGFRA^low/–^PDPN^+^CADM1^+^ and PDGFRA^low/–^PDPN^+^CADM1^–^ cells were sorted by flow cytometry and cultured for 7–10 days as previously described.^97^ Briefly, 5 × 10^5^ cells were resuspended in 5 μL of Matrigel (BD) on ice and then aspirated into a micropipette (Drummond Scientific, 5-000-2010). A small incision was made near the kidney pole to separate the capsule from the renal parenchyma. Matrigel with cells was injected into the kidney pocket. Eight weeks after transplantation, grafts were dissected and fixed in 4% paraformaldehyde at 4 °C for 12 h, decalcified in 10% EDTA at room temperature for 3 days and then dehydrated in 30% sucrose at 4 °C overnight. Grafts were then cryosectioned at 10 μm and stained by Movat Pentachrome Staining Kit (ScyTek, MPS-1) to demonstrate bone and cartilage differentiation. Immunostaining of collagen I and II were also performed on adjacent sections (see below).

### Immunofluorescent staining

Slides containing renal subcapsular graft cryosections were blocked (10% horse serum and 0.1% Triton X-100 in PBS) at room temperature for 1 h and stained with anti-collagen I (Abcam, ab34710, 1:500) and anti-collagen II (Abcam, ab185430, 1:500) antibodies at 4 °C overnight. After washing in PBS (3 × 10 min), anti-Rabbit Alexa Fluor 555 (Invitrogen, A31572, 1:500) and anti-Mouse Alexa Fluor 647 (Invitrogen, A31571, 1:500) secondary antibodies were incubated for 1 h at room temperature. After washing in PBS (3 × 10 min), slides were mounted with ProLong™ Gold Antifade Mountant with DAPI (Invitrogen, P36931). For long bone and calvarial staining, the following antibodies were used: anti-PDPN (eBioscience, 17-9381-41, 1:50), anti-CADM1 (abcam, ab3910, 1:100), anti-FOXP1 (Sigma-Aldrich, ABE68, 1:100), anti-FOXP2 (Abcam, ab16046, 1:200), anti-Rabbit Alexa Fluor 555 (Invitrogen, A31572,1:500) and anti-Rat Alexa Fluor 647 (Invitrogen, A21472, 1:500). Images were acquired with Olympus fluorescence inverted microscope (IX73) and analyzed by ImageJ software.

### scRNA-seq

Samples from different stages were harvested and live cells were sorted based on 7-AAD staining (90%–95% viability after sorting). Cells were resuspended at 1 × 10^3^ cells/mL and loaded on Chromium Controller to obtain single cells (10× Genomics). For scRNA-seq library construction, Chromium Single cell 3’ Library and Gel Bead Kit V2 (10× Genomics, PN120237) was used to generate single cell gel beads in emulsion (GEM). The captured cells were lysed, and the released RNA was reverse-transcribed with primers containing poly-T, barcode, unique molecular Identifiers (UMIs) and read 1 primer sequence in GEMs. Barcoded cDNA was purified and amplified by PCR. The adapter ligation reaction was performed to add sample index and read 2 primer sequence. After quality control, the libraries were sequenced on Illumina Hiseq X Ten platform in 150 bp pair-ended manner (Berry Genomics Corporation, Beijing, China).

### Processing of scRNA-seq data

Sequencing data from 10× Genomics were processed with *CellRanger* (version 3.0.1) for demultiplexing, barcode processing and single-cell 3′ gene counting. Human genome reference (GRCh38) was used for sequence alignment. Only confidently mapped, non-PCR duplicates with valid barcodes and UMIs were used to generate the gene-barcode matrix. For quality control, only cells with more than 1000 genes and less than 10% of mitochondrial gene were retained for downstream analysis. Cell doublets were removed using *Scrublet* software implemented in python^98^ (https://github.com/AllonKleinLab/scrublet). Briefly, we computed doublet score for each cell by applying *Scrublet* function to each 10× dataset. Then we estimated the number of expected doublets (*N*) with multiplet rates (based on the number of cells recovered) provided by 10× Genomics guideline. Top *N* of cells ranked by doublet scores were determined as doublets (Supplementary information, Figs. S1a and S6a). To correct batch effects among different samples, we applied CCA method implemented in Seurat for dataset integration.^45^ The union of the top 2000 genes with the highest dispersion for each dataset was taken to identify anchors using the *FindIntegrationAnchors* function and calculate 30 dimensionalities. We then applied *IntegrateData* function to generate integrated expression matrix, which was used for dimensionality reduction and clustering subsequently. To exclude karyotype abnormalities in human embryos, we applied CNV estimation for single cells in 10× datasets from a previous study.^44^ Briefly, we downloaded the expression matrix of non-malignant cells (T cells) and malignant cells as reference cells for the estimation of CNVs. We sampled 100 cells for each 10× dataset and combined them with reference cells to calculate initial CNVs and final CNVs. The CNV correlation score of each single-cell was computed and visualized by heatmap (Supplementary information, Fig. S1b).

### Dimensionality reduction and clustering

To reduce the variation in cell proliferation status that might interfere with single cell analysis, we used the previously reported G1/S and G2/M phase-specific genes to compute scores of S phase and G2M phase, as well as estimate cell cycle status.^99^ We scaled the integrated data with regressing the *S.Score* and *G2M.Score*, and calculated the top 30 PCs. For dimensionality reduction, we performed UMAP on whole datasets, and used Diffusion map and PCA to visualize the subset of datasets (Supplementary information, Table S3). t-Distributed Stochastic Neighbor Embedding (t-SNE) was applied to visualize the relationships between cell clusters at pseudo-bulk level. For clustering, improved graph-based clustering of the integrated dataset was performed using louvain algorithm after constructing the Shared Nearest Neighbor (SNN) graph. The resolution parameters were set to 0.2 (Supplementary information, Table S3). To ensure the robustness of clustering, we randomly subsampled 1000 cells from each dataset, and re-processed as previous steps and parameters. The newly identified clusters showed an average assignment of 80% to clusters identified in the whole dataset.

### Species comparative analysis

For comparative analysis between human and mouse datasets, the expression data matrix of mouse E11.5 and E15.5 from GSE142425 were collected.^70^ To ensure the comparability, the stage correspondences were identified^100^ and the mouse datasets were processed by the same steps as human datasets, including dimension reduction and clustering. *SciBet* R package (version 1.0)^71^ was used to compare cell subsets identified in limb buds and long bones. We used the expression matrix of human cells as reference dataset and calculated the mean expression values of marker genes across cells with identical cell types. Multinomial models were then built and the query mouse dataset were re-annotated. Sankey plot with *ggalluvial* R package was applied to visualize the matching degree of predicted mouse cell type to the human reference.

### Differential expression analysis

Non-parametric Wilcoxon rank sum test was performed to find DEGs among individual clusters. DEGs were filtered by fold change of more than 2 and cell fraction of more than 20%. DEGs with *P* value adjusted by Benjamini–Hochberg less than 0.01 were considered to be significant (Supplementary information, Table S1).

### Single-cell regulatory network analysis

The analysis of single-cell gene regulatory network was performed using the *SCENIC* package.^64^ A standard pipeline implemented in R can be found in https://github.com/aertslab/SCENIC. The expression matrix was loaded onto *GENIE3* for building the initial co-expression gene regulatory networks (GRN). The regulon data was then analyzed using the *RcisTarget* package to create TF motifs using hg19-tss-centered-10kb (for human) and mm9-tss-centered-10kb (for mouse) database. The regulon activity scores were calculated with AUC by the *AUCell* package. Significant regulons enriched in different clusters were calculated by two-sided unpaired *t*-test implemented in Limma R package (version 3.38.3) (Supplementary information, Table S2). The mean regulon activity scores for each cluster were calculated and visualized by heatmap. Predicted target genes of regulon were ranked by *Genie3Weight* value and filtered by normalized enrichment score (NES) of binding motifs (greater than 3). The transcriptional network of TF and predicted target genes was visualized by *Cytoscape* (version 3.6). Edges indicated the *Genie3Weights* and Node size indicated the number of motifs.

### Reconstructing single-cell trajectory

Single-cell trajectory was analyzed by R package *Slingshot* (version 1.0.0), which infers trajectory by fitting principal curves based on given cell embeddings.^84^ After specifying the start or end cluster of the trajectory, cells were projected onto the curve to assign their developmental pseudotime. Specifically, we computed the diffusion map embeddings of LBDMCs, eSSPCs, osteoprogenitors and two subsets of chondrocytes to infer osteo-chondrogenic trajectory. The diffusion components 1 and 3 were used as the input to *Slingshot* (Fig. 3d), and LBDMC was set as start cluster. For calvarial osteogenesis trajectory, we re-computed the UMAP embedding of NCs, mig_NCs, NCDCs, osteoprogenitors and two subsets of PMSCs, and used UMAP component 1 and 2 as the input to *Slingshot*. The osteoprogenitor subset was set as end cluster (Fig. 6e). To investigate temporally expressed genes changing in a continuous manner over pseudotime, *GAM* function implemented in gam R package was used to find pattern genes along the trajectories. For identification of major patterns, top 200 genes with the most significant time-dependent model fit were retained, and expressions of these genes were smoothed over 20 adjacent cells. To quantify the connectivity of clusters within single-cell graph, the PAGA method implemented in Scanpy (version 1.4.3)^101^ was used to generate the abstracted graph.

### RNA velocity

RNA velocity^58^ was used for pseudo-time analysis in the integrated dataset of limb buds and long bones (Fig. 1f), as well as OCLC subsets (Fig. 3c). The spliced and un-spliced reads were quantified by the *velocyto* (version 0.17.11) python package with human genome reference. The output loom file was analyzed for velocities of each gene following the pipeline of *scvelo* python package (version 0.1.25).^102^ Count matrix was filtered by top 2000 highly variable genes and first- and second-order moments were computed for each cell with nearest neighbor set to 30.

### TACS

We adopted TACS as previously described to evaluate cell distribution along selected query genes.^76^ For each cell, average expression of the top 100 correlated genes was set as the expression score of the query gene. *Stat_density2d* function implemented in *ggplot2* package was used for visualization. Threshold for partitioning was set to zero.

### Gene functional annotation analysis

GO enrichment analysis was performed for DEGs using *clusterProfiler* package.^103^ The significant DEGs were used as input to *compareCluster* function and ontology was set to the BP (biological process). The *P* values of enriched GO terms were adjusted by *Benjamini-Hochberg* method and terms were filtered by setting *pvalueCutoff* to 0.05. *Simplify* function was performed to remove redundancy of the enriched GO terms.

### Gene set analysis

GSVA was performed using the *GSVA* R package (version 1.30.0).^104^ We selected gene sets of curated signaling pathways from the MSigDB Database (v7.0, https://www.gsea-msigdb.org) to identify pathways enriched in different limb mesenchymal subsets. The gene-by-cell matrix was converted to gene-set-by-cell matrix and GSVA scores were computed for gene sets with a minimum of 5 detected genes. Significant pathways enriched in different clusters were calculated by two-sided unpaired *t*-test implemented in Limma R package (version 3.38.3).

### Surface markers and TFs

Surface marker and TF lists were downloaded from the in silico human surfaceome (http://wlab.ethz.ch/surfaceome/)^105^ and HumanTFDB3.0 (http://bioinfo.life.hust.edu.cn/HumanTFDB/) database websites (Supplementary information, Table S4).

### Statistics and reproducibility

Values in scatter plots were presented as means ± SEM. Statistical analyses were performed using R and SPSS. The statistical significance of differences was determined using one-way ANOVA with LSD multiple comparison tests (Fig. 4e; Supplementary information, Fig. S4d). Wilcoxon signed rank test was used to determine the statistical significance of differences for gene expression (2^–ΔΔCt^) analyses (Fig. 5c; Supplementary information, Figs. S4h and S5f, h). For scRNA-seq experiments, we analyzed three independent embryos for limb buds (5 WPC), three independent embryos for long bones (8 WPC), two independent embryos for calvaria (8 WPC), and one embryo for eSSPC phenotypic marker validation (Supplementary information, Fig. S5a–c). For functional experiments, we analyzed two independent embryos for H&E staining and immunostaining (Figs. 1a, 3i, j, 4b; Supplementary information, Figs. S4b, S6d), three independent embryos for CFU-F colony and sphere formation analyses (Fig. 4c–e; Supplementary information, Fig. S4c, d), two independent embryos for PDGFRA^+^ cell colony formation analysis (Supplementary information, Fig. S4e, f), three independent clones for clonal differentiation analyses (Fig. 5a–c; Supplementary information, Fig. S5g, h), three independent embryos for nonclonal differentiation analyses (Supplementary information, Fig. S5e, f), three independent embryos for renal subcapsular transplantation of eSSPCs (Fig. 5d), and one embryo for renal subcapsular transplantation of PDGFRA^low/–^PDPN^+^CADM1^–^ cells (Supplementary information, Fig. S5i).

## Supplementary information

Supplementary information, Figure S1

Supplementary information, Figure S2

Supplementary information, Figure S3

Supplementary information, Figure S4

Supplementary information, Figure S5

Supplementary information, Figure S6

Supplementary information, Table S1

Supplementary information, Table S2

Supplementary information, Table S3

Supplementary information, Table S4

Supplementary information, Table S5

## Data Availability

The accession number for the human scRNA-seq data reported in this paper is GEO: GSE143753. All other relevant data are available from the corresponding authors upon request. The accession number for the count matrices of mouse datasets used in this paper is GSE142425.^70^
