# Supplementary material for: Dissecting human embryonic skeletal stem cell ontogeny by single-cell transcriptomic and functional analyses
Source: Cell Res. 2021 Jan 20;31(7):742–57. doi: 10.1038/s41422-021-00467-z (PMC8249634; doi:10.1038/s41422-021-00467-z)
Supplement: Supplementary file 1 — Supplementary information, Figure S1 [file 41422_2021_467_MOESM1_ESM.pdf]

Figure S1

a

Stage	Sample	Dpf[b]	Tissue	Strategy		Gender	Cell Number	Doublets	Cell Number (QC[c])	Gene number	UMI number	Perc.mito[d]
5 WPC[a]	Embryo 1	30	Limb bud	scRNA-seq	7AAD⁻ CD235A⁻	Male	6,248	315	5,300	3,443	13,741	2.5%
	Embryo 2	36	Limb bud	scRNA-seq		Female	8,675	606	7,351	3,508	13,079	2.8%
	Embryo 3	36	Limb bud	scRNA-seq		Male	9,119	670	7,239	3,119	10,759	2.2%
8 WPC	Embryo 4	49	Long bone	scRNA-seq		Female	4,786	185	4,375	2,253	7,159	1.5%
	Embryo 5	55	Long bone	scRNA-seq		Male	6,645	356	5,745	2,386	8,760	1.9%
	Embryo 6	55	Long bone	scRNA-seq		Female	6,431	334	5,560	1,955	6,247	2.3%
	Embryo 7	56	Long bone	scRNA-seq	PDGFRA$^{low/-}$ PDPN^{+}CADM1^{+}	Female	334	NA	314	1,739	4,741	1.4%
	Embryo 8-10	55	Long bone	Nonclonal culture								
	Embryo 11-12	55	Long bone	Clonal culture								
	Embryo 13-15	56	Long bone	Clonal culture								
	Embryo 16-19	56	Long bone	Transplant								
	Embryo 20-21	36	Limb bud	H&E staining								
	Embryo 22-23	56	Long bone	H&E, IF[e] staining								

[a]WPC: weeks post conception; [b]Dpf: days post fertilization;
[c]QC: quality control; [d]Pec.mito: percentage of mitochondrial genes
[e]IF: immunofluorescence

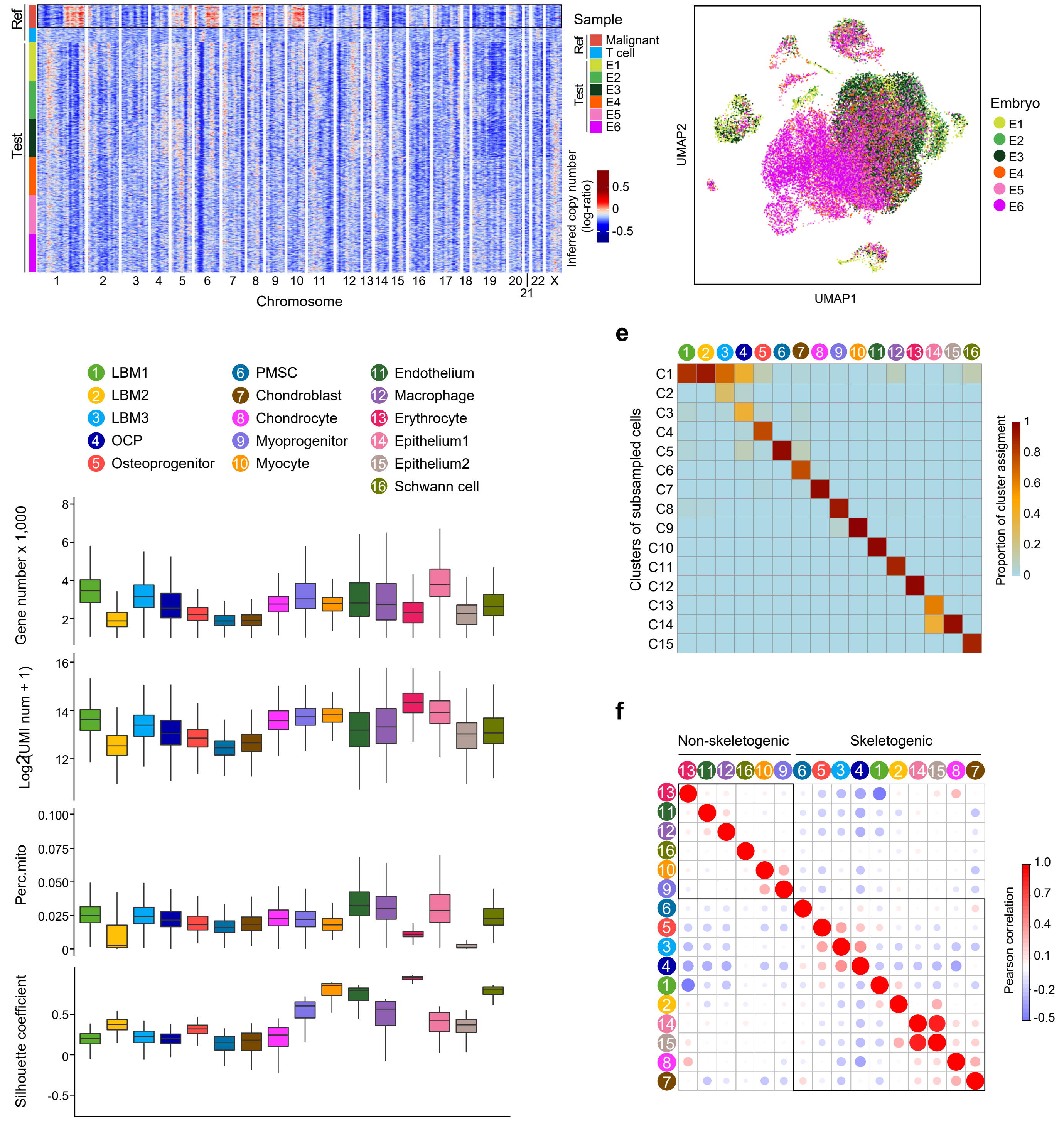

Supplementary Fig. 1. Sample information and data quality control

a, Table summary of human embryonic limb bud and long bone samples and detailed scRNA-seq information.

b, CNV scores inferred from transcriptomes of tumor cells, normal T cells (reference cell type) and 100 randomly selected cells from the 6 embryos analyzed by scRNA-seq (test cells). Red: amplifications; Blue: deletions.

c, UMAP visualization of the 6 embryos analyzed by scRNA-seq. These included 5 WPC limb buds (E1-3) and 8 WPC long bones (E4-6).

d, Boxplot showing the number of detected genes, log-transformed UMI counts, percentage of mitochondrial genes and Silhouette coefficient for each subset.

e, Assessment of the 15 clusters from 6,000 randomly subsampled cells (1000 cells from each embryo) to the 16 subsets annotated in Fig. 1c.

f, Pearson correlation analysis showing the relationship among the 16 subsets. Hierarchical clustering according to Pearson correlation distinguished skeletogenic (clusters 1-8, 14, 15) and non-skeletogenic subsets (clusters 9-13 and 16).
