# Supplementary material for: Dissecting human embryonic skeletal stem cell ontogeny by single-cell transcriptomic and functional analyses
Source: Cell Res. 2021 Jan 20;31(7):742–57. doi: 10.1038/s41422-021-00467-z (PMC8249634; doi:10.1038/s41422-021-00467-z)
Supplement: Supplementary file 2 — Supplementary information, Figure S2 [file 41422_2021_467_MOESM2_ESM.pdf]

Figure S2

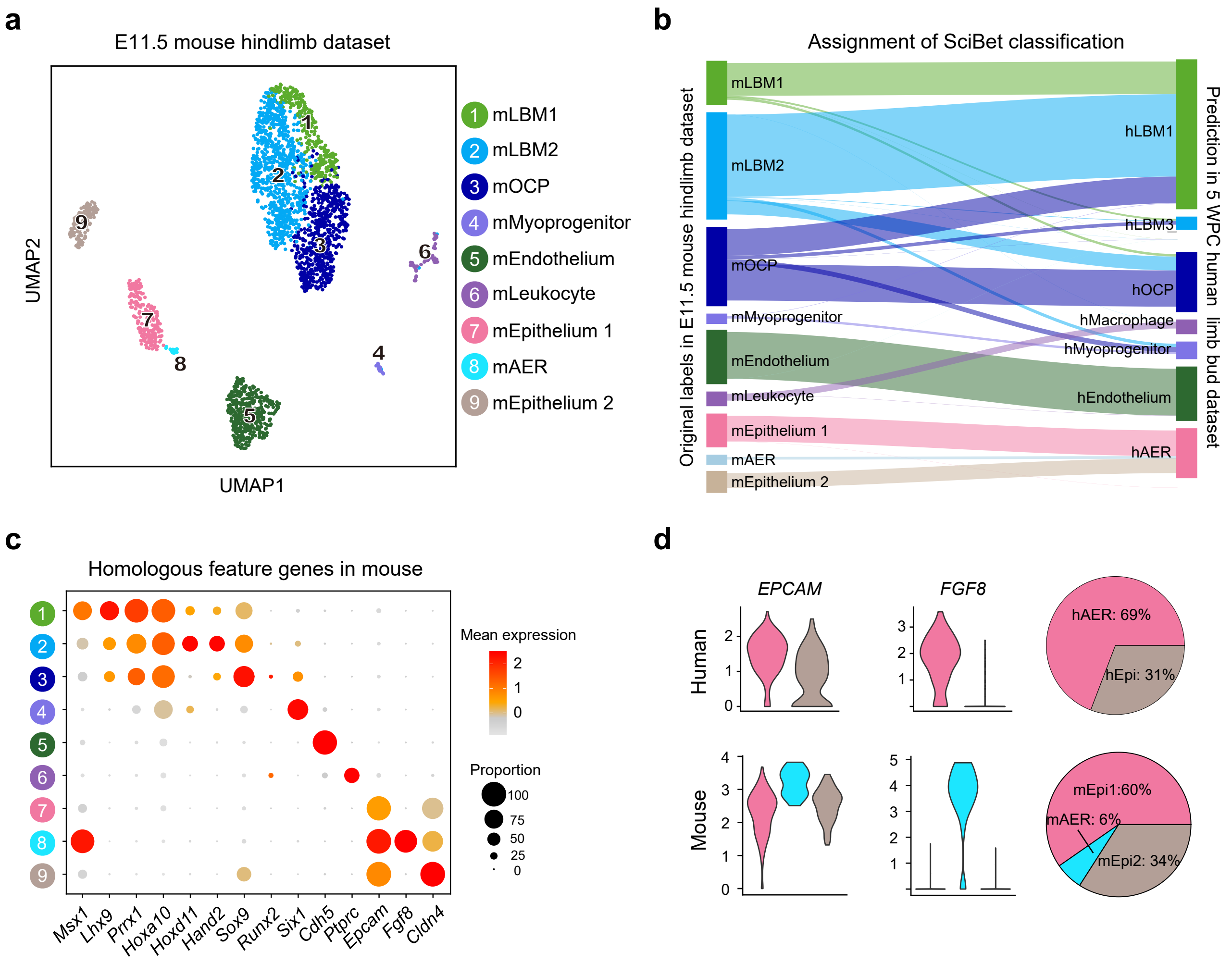

Supplementary Fig. 2. Characterization of E11.5 mouse hindlimb bud mesenchyme and epithelium

a, UMAP visualization of 9 cell subsets in E11.5 mouse hindlimb bud dataset. Expression matrix was re-processed and cells were clustered according to the expression of homologous feature genes in human limb bud.

b, Sankey diagram for assigning E11.5 mouse hindlimb bud subsets to 5 WPC human limb bud datasets.

c, Dot plots of mean expression of homologous feature genes in E11.5 mouse hindlimb bud subsets.

d, Violin plots (left) showing the gene expression of EPCAM and FGF8 in human and mouse epithelial subsets. Pie charts (right) showing the proportions of each epithelial subset.
