# Supplementary material for: Dissecting human embryonic skeletal stem cell ontogeny by single-cell transcriptomic and functional analyses
Source: Cell Res. 2021 Jan 20;31(7):742–57. doi: 10.1038/s41422-021-00467-z (PMC8249634; doi:10.1038/s41422-021-00467-z)
Supplement: Supplementary file 3 — Supplementary information, Figure S3 [file 41422_2021_467_MOESM3_ESM.pdf]

Figure S3

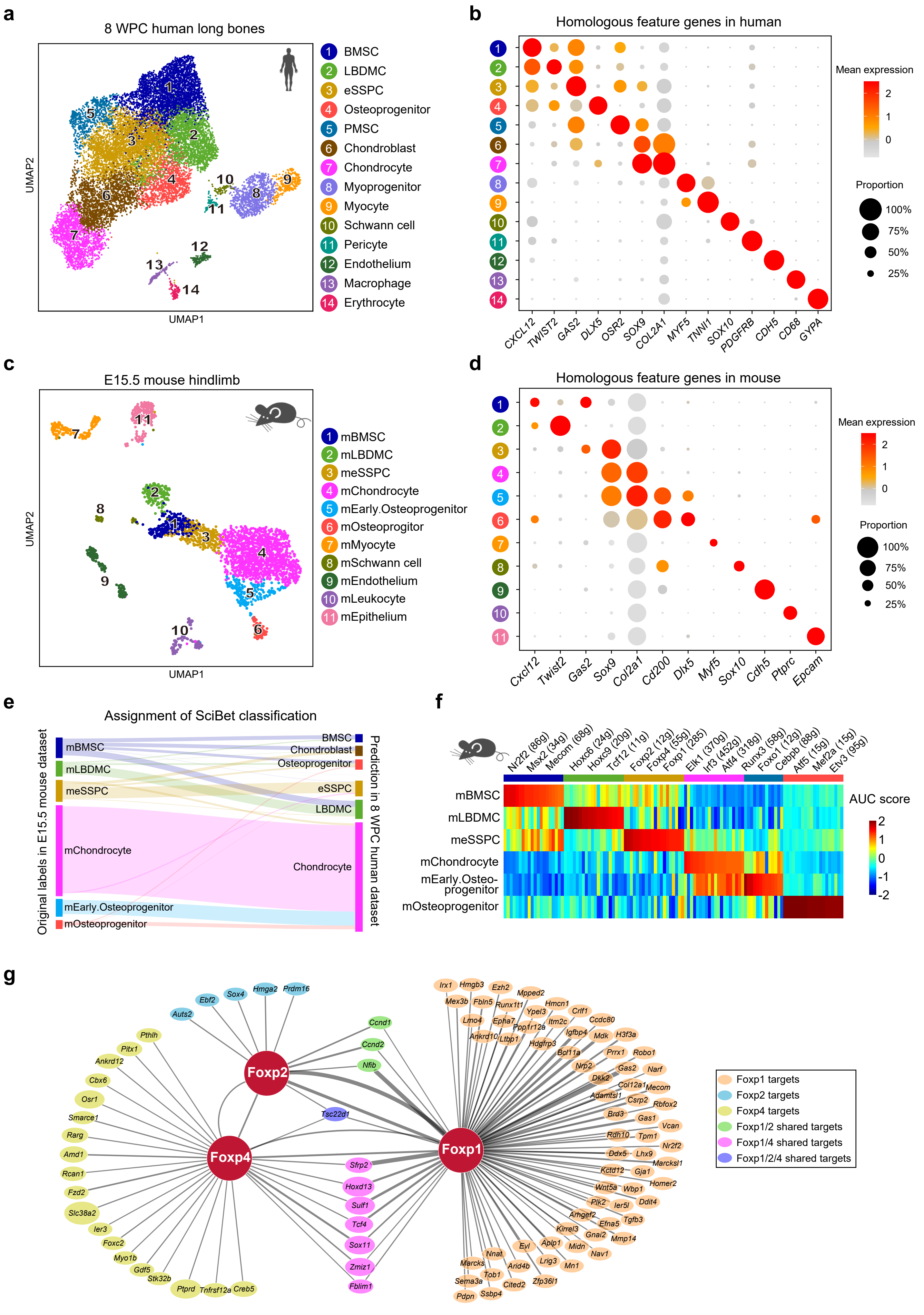
a
8 WPC human long bones
UMAP1
UMAP2
1 BMSC
2 LBDMC
3 eSSPC
4 Osteoprogenitor
5 PMSC
6 Chondroblast
7 Chondrocyte
8 Myoprogenitor
9 Myocyte
10 Schwann cell
11 Pericyte
12 Endothelium
13 Macrophage
14 Erythrocyte
b
Homologous feature genes in human
CXCL12 TWIST2 GAS2 DLX5 OSR2 SOX9 COL2A1 MYF5 TNNI1 SOX10 PDGFRB CDH5 CD68 GYPA
Mean expression
2
1
0
Proportion
100%
75%
50%
25%
c
E15.5 mouse hindlimb
UMAP1
UMAP2
1 mBMSC
2 mLBDMC
3 meSSPC
4 mChondrocyte
5 mEarly.Osteoprogenitor
6 mOsteoprogitor
7 mMyocyte
8 mSchwann cell
9 mEndothelium
10 mLeukocyte
11 mEpithelium
d
Homologous feature genes in mouse
Cxcl12 Twist2 Gas2 Sox9 Col2a1 Cd200 Dlx5 Myf5 Sox10 Cdh5 Ptprc Epcam
Mean expression
2
1
0
Proportion
100%
75%
50%
25%
e
Assignment of SciBet classification
Original labels in E15.5 mouse dataset
Prediction in 8 WPC human dataset
mBMSC
mLBDMC
meSSPC
mChondrocyte
mEarly.Osteoprogenitor
mOsteoprogenitor
BMSC
Chondroblast
Osteoprogenitor
eSSPC
LBDMC
Chondrocyte
f
Nr2f2 (86g) Msx2 (34g) Mecom (68g) Hoxc6 (24g) Hoxc9 (20g) Tcf12 (11g) Foxp2 (12g) Foxp4 (55g) Foxp1 (285) Elk1 (370g) Irf3 (452g) Atf4 (318g) Runx3 (58g) Foxo1 (12g) Cebpb (88g) Atf5 (15g) Mef2a (15g) Etv3 (95g)
mBMSC
mLBDMC
meSSPC
mChondrocyte
mEarly.Osteo-
progenitor
mOsteoprogenitor
AUC score
2
1
0
-1
-2
g
Foxp2
Foxp4
Foxp1
Foxp1 targets
Foxp2 targets
Foxp4 targets
Foxp1/2 shared targets
Foxp1/4 shared targets
Foxp1/2/4 shared targets
Ebf2 Sox4 Hmga2 Prdm16 Auts2
Ccnd1 Ccnd2 Nfib Tsc22d1
Pthlh Pitx1 Ankrd12 Cbx6 Osr1 Smarce1 Rarg Amd1 Rcan1 Fzd2 Slc38a2 Ier3 Foxc2 Myo1b Gdf5 Stk32b Ptprd Tnfrsf12a Creb5
Sfrp2 Hoxd13 Sulf1 Tcf4 Sox11 Zmiz1 Fblim1
Irx1 Hmgb3 Ezh2 Mpped2 Mex3b Fbln5 Runx1t1 Hmcn1 Ypel3 Crlf1 Lmo4 Epha7 Ppp1r12a Itm2c Ccdc80 Ankrd10 Ltbp1 Igfbp4 Mdk H3f3a Hdgfrp3 Bcl11a Prrx1 Robo1 Nrp2 Gas2 Narf Dkk2 Col12a1 Mecom Adamtsl1 Csrp2 Rbfox2 Brd3 Gas1 Vcan Rdh10 Tpm1 Nr2f2 Ddx5 Lhx9 Marcksl1 Kctd12 Gja1 Homer2 Wnt5a Wbp1 Plk2 Ier5l Ddit4 Arhgef2 Efna5 Tgfb3 Kirrel3 Gnai2 Mmp14 Nav1 Evl Aplp1 Midn Lrig3 Mn1 Nnat Arid4b Marcks Tob1 Cited2 Zfp36l1 Sema3a Pdpn Ssbp4

Supplementary Fig. 3. Cross-species comparison between human and mouse embryonic long bones during POC formation

a, UMAP plot of the 14 subsets in 8 WPC human long bones.

b, Dot plots showing the expression of human homologous feature genes in the 14 subsets indicated in **(a)**.

c, UMAP plot of the 11 subsets in re-processed E15.5 mouse hindlimb dataset.

d, Dot plots showing the expression of mouse homologous feature genes in the 11 mouse hindlimb subsets indicated in **(c)**.

e, Sankey diagram for assigning mouse E15.5 hindlimb datasets to human 8 WPC long bone datasets.

f, Heatmap showing the AUC scores of regulons enriched in mouse OCLC subsets. Z-score (column scaling) was calculated. Representative regulons were shown on the top.

g, The Foxp1/2/4 regulon networks in mouse OCLC subsets. Lines thickness indicated the level of GENIE3 weights. Dot size indicated the number of enriched TF motifs.
