# Supplementary material for: Dissecting human embryonic skeletal stem cell ontogeny by single-cell transcriptomic and functional analyses
Source: Cell Res. 2021 Jan 20;31(7):742–57. doi: 10.1038/s41422-021-00467-z (PMC8249634; doi:10.1038/s41422-021-00467-z)
Supplement: Supplementary file 4 — Supplementary information, Figure S4 [file 41422_2021_467_MOESM4_ESM.pdf]

Figure S4

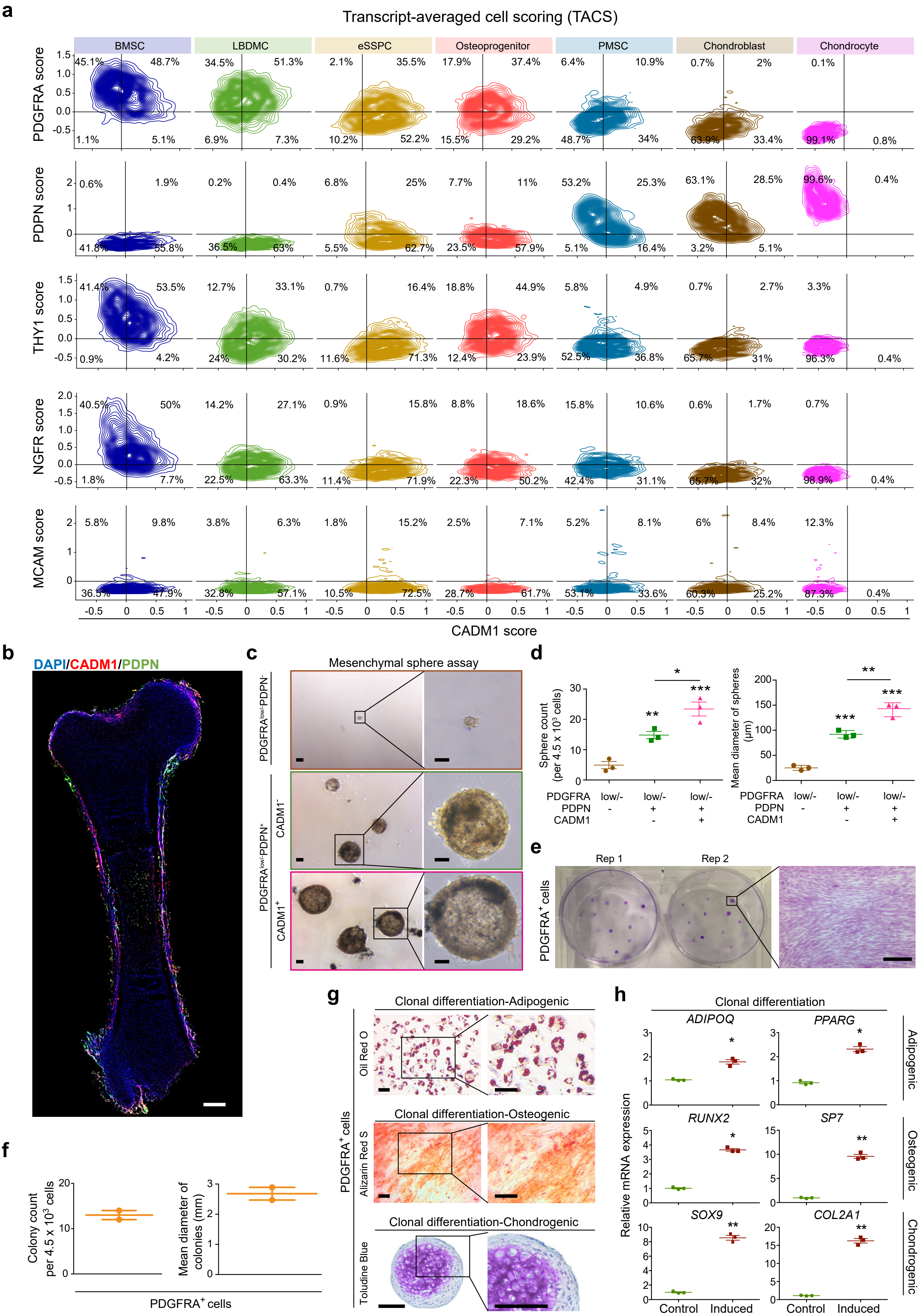

a
Transcript-averaged cell scoring (TACS)
BMSC
LBDMC
eSSPC
Osteoprogenitor
PMSC
Chondroblast
Chondrocyte
PDGFRA score
PDPN score
THY1 score
NGFR score
MCAM score
CADM1 score
b
DAPI/CADM1/PDPN
c
Mesenchymal sphere assay
PDGFRAlow/-PDPN-
PDGFRAlow/-PDPN+
CADM1-
CADM1+
d
Sphere count (per 4.5 x 10³ cells)
Mean diameter of spheres (μm)
PDGFRA
PDPN
CADM1
e
Rep 1
Rep 2
PDGFRA+ cells
f
Colony count per 4.5 x 10³ cells
Mean diameter of colonies (mm)
PDGFRA+ cells
g
Clonal differentiation-Adipogenic
Oil Red O
Clonal differentiation-Osteogenic
Alizarin Red S
Clonal differentiation-Chondrogenic
Toludine Blue
PDGFRA+ cells
h
Clonal differentiation
ADIPOQ
PPARG
RUNX2
SP7
SOX9
COL2A1
Relative mRNA expression
Adipogenic
Osteogenic
Chondrogenic
Control
Induced

Supplementary Fig. 4. Further in silico and functional analyses of eSSPCs and PDGFRA+ cells
a, TACS plots showing the distribution of each OCLC subset between indicated surface marker pairs. Contours outlined regions of increasingly higher cell density. Cell frequencies were shown on the plots.
b, Representative immunofluorescent image of 8 WPC human femur section stained with DAPI (blue), CADM1 (red) and PDPN (green) (n=2 embryos). Scale bar: 200 μm.
c, Representative images showing the mesenchymal spheres formed by the 3 populations sorted as in Fig. 4c (left), with magnified views (right). Scale bars: 25 μm.
d, Quantifications of the number (left) and mean diameter (right) of mesenchymal spheres (n=3 embryos). The statistical significance of differences was determined using one-way ANOVA with multiple comparison tests (LSD). * $P < 0.05$; ** $P < 0.01$; *** $P < 0.001$. Error bars indicated SEM.
e, Representative crystal violet staining of CFU-F colonies generated by flow cytometrically sorted $PDGFRA^{+}$ cells from two independent 8 WPC long bones. Magnified images of the boxed areas were shown on the right. Scale bar: 25 μm.
f, Quantifications of the number (left) and mean diameter (right) of CFU-F colonies generated by flow cytometrically sorted $PDGFRA^{+}$ cells (n=2 embryos). Error bars indicated SEM.
g and **h**, Representative oil red O (top), alizarin red (middle) and toluidine blue (bottom) staining after adipogenic, osteogenic and chondrogenic differentiation of clonally expanded $PDGFRA^{+}$ cells (**g**) with qPCR quantifications (**h**). Magnified images of the boxed areas were shown on the right (n=2 embryos). Scale bars: 200 μm. The statistical significance of differences was determined using Wilcoxon signed rank test. * $P < 0.05$; ** $P < 0.01$. Error bars indicated SEM.
