# Supplementary material for: Dissecting human embryonic skeletal stem cell ontogeny by single-cell transcriptomic and functional analyses
Source: Cell Res. 2021 Jan 20;31(7):742–57. doi: 10.1038/s41422-021-00467-z (PMC8249634; doi:10.1038/s41422-021-00467-z)
Supplement: Supplementary file 5 — Supplementary information, Figure S5 [file 41422_2021_467_MOESM5_ESM.pdf]

Figure S5

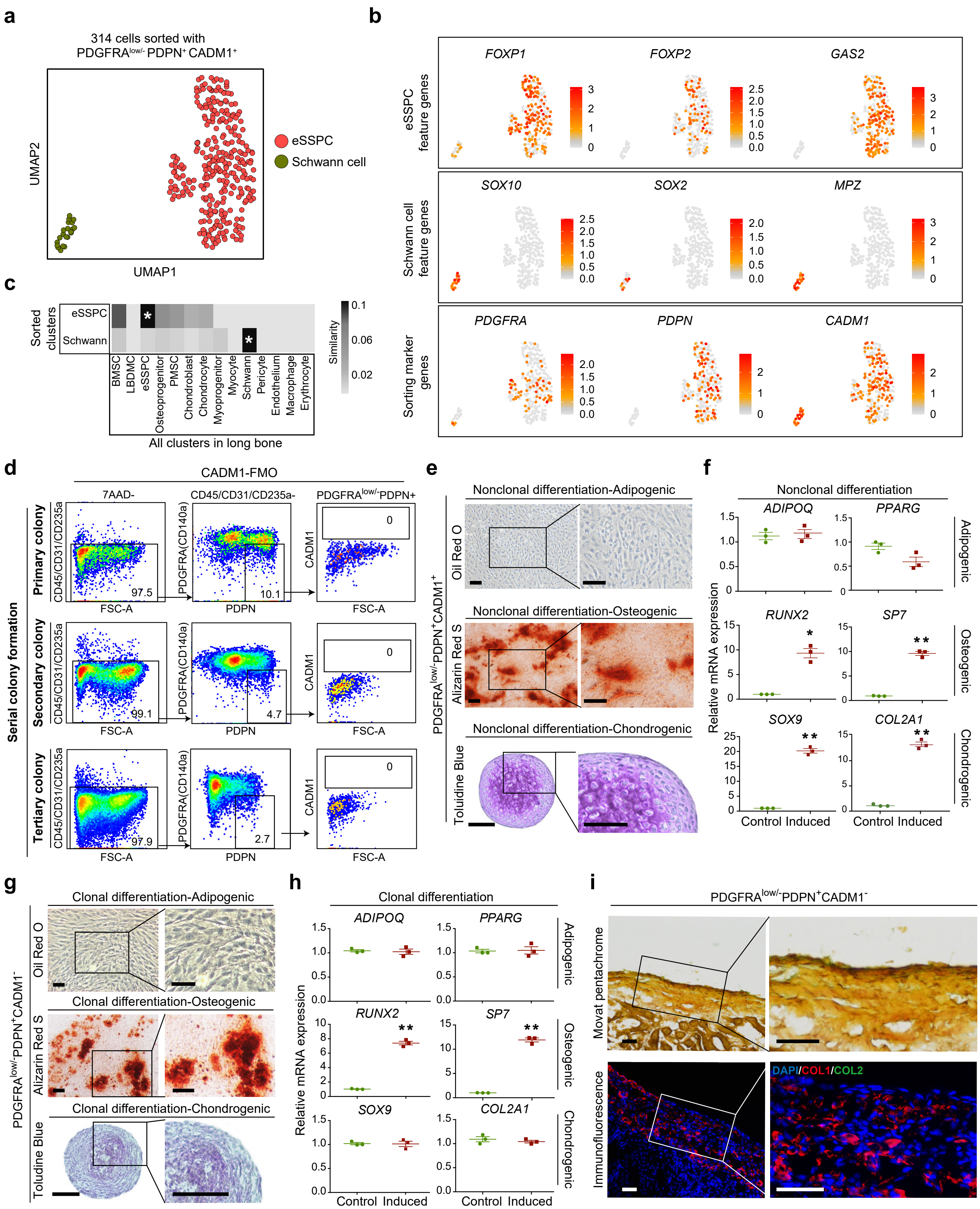

a
314 cells sorted with PDGFRA^low/- PDPN+ CADM1+
UMAP2
UMAP1
eSSPC
Schwann cell
b
eSSPC feature genes
FOXP1
FOXP2
GAS2
Schwann cell feature genes
SOX10
SOX2
MPZ
Sorting marker genes
PDGFRA
PDPN
CADM1
c
Sorted clusters
eSSPC
Schwann
Similarity
BMSC
LBDMC
eSSPC
Osteoprogenitor
PMSC
Chondroblast
Chondrocyte
Myoprogenitor
Myocyte
Schwann
Pericyte
Endothelium
Macrophage
Erythrocyte
All clusters in long bone
d
CADM1-FMO
7AAD-
CD45/CD31/CD235a-
PDGFRA^low/-PDPN+
Serial colony formation
Primary colony
Secondary colony
Tertiary colony
FSC-A
PDPN
PDGFRA(CD140a)
CADM1
e
Nonclonal differentiation-Adipogenic
Nonclonal differentiation-Osteogenic
Nonclonal differentiation-Chondrogenic
Oil Red O
Alizarin Red S
Toluidine Blue
PDGFRA^low/-PDPN+CADM1+
f
Nonclonal differentiation
ADIPOQ
PPARG
RUNX2
SP7
SOX9
COL2A1
Relative mRNA expression
Adipogenic
Osteogenic
Chondrogenic
Control Induced
g
Clonal differentiation-Adipogenic
Clonal differentiation-Osteogenic
Clonal differentiation-Chondrogenic
Oil Red O
Alizarin Red S
Toludine Blue
PDGFRA^low/-PDPN+CADM1-
h
Clonal differentiation
ADIPOQ
PPARG
RUNX2
SP7
SOX9
COL2A1
Relative mRNA expression
Adipogenic
Osteogenic
Chondrogenic
Control Induced
i
PDGFRA^low/-PDPN+CADM1-
Movat pentachrome
Immunofluorescence
DAPI/COL1/COL2

Supplementary Fig. 5. Validation of eSSPC phenotypic markers by scRNA-seq and further characterizations of PDGFRA$^{low/-}$PDPN^{+}CADM1$^{+/-}$ cells

a, Distribution of 314 PDGFRA$^{low/-}$PDPN^{+}CADM1^{+} cells sorted from 8 WPC long bone. eSSPC and Schwann cell subsets were visualized by UMAP (n=1 embryo).

b, UMAP plots showing the expression of eSSPC feature genes (top), Schwann cell feature genes (middle) and sorting marker genes (bottom) in the cells of Fig.S5a.

c, Heatmap showing the Jaccard similarity of eSSPC and Schwann cell subsets in Fig.S5a and cell subsets of 8 WPC long bone datasets in Fig.S3a. Jaccard similarity was calculated using DEGs from different datasets. Asterisks indicated subsets with similarity > 0.1.

d, Fluorescence-minus-one (FMO) controls for eSSPC gating strategy in serial colony formation assay (Fig. 5a).

e and **f**, Representative oil red O (top), alizarin red S (middle) and toluidine blue (bottom) staining after adipogenic, osteogenic and chondrogenic differentiation of nonclonally expanded eSSPCs (PDGFRA$^{low/-}$PDPN^{+}CADM1^{+}) (**e**) with qPCR quantifications (**f**) (n=3 embryos). Magnified images of the boxed areas were shown on the right. Scale bars: 200 μm.

g and h, Representative oil red O (top), alizarin red S (middle) and toluidine blue (bottom) staining after adipogenic, osteogenic and chondrogenic differentiation of clonally expanded PDGFRA$^{low/-}$PDPN^{+}CADM1^{-} cells (**g**) with qPCR quantifications (**h**) (n=3 clones). Magnified images of the boxed areas were shown on the right. Scale bars: 200 μm.

i, Renal subcapsular transplantation of PDGFRA$^{low/-}$PDPN^{+}CADM1^{-} cells. Movat pentachrome staining (top, bone and fibrous tissue: yellow) and immunofluorescent staining images (bottom, DAPI: blue, collagen I (COL I): red, collagen II (COL I): green) were shown (n=4 grafts from one embryo). Magnified images of the boxed areas were shown on the right. Scale bars: 50 μm.

The statistical significance of differences was determined using Wilcoxon signed rank test.

* $P < 0.05$; ** $P < 0.01$. Error bars indicated SEM.
