# Supplementary material for: Dissecting human embryonic skeletal stem cell ontogeny by single-cell transcriptomic and functional analyses
Source: Cell Res. 2021 Jan 20;31(7):742–57. doi: 10.1038/s41422-021-00467-z (PMC8249634; doi:10.1038/s41422-021-00467-z)
Supplement: Supplementary file 6 — Supplementary information, Figure S6 [file 41422_2021_467_MOESM6_ESM.pdf]

Figure S6

a

Stage	Sample	Dpf[a]	Tissue	Strategy	Gender	Cell Number	Doublets	Cell Number (QC[b])	Gene number	UMI number	Perc.mito[c]
8 WPC	Embryo 24	56	Calvarial bone	scRNA-seq on 7AAD⁻CD235A⁻ cells	Male	3,786	338	2,932	3,126	14,860	2.7%
	Embryo 25					6,476	116	4,355	4,330	17,899	6.5%
	Embryo 26-27	56	Calvarial bone	IF[e] staining							

[a]WPC: weeks post conception; [b]Dpf: days post fertilization; [c]QC: quality control; [d]Pec.mito: percentage of mitochondrial genes; [e]IF: immunofluorescence

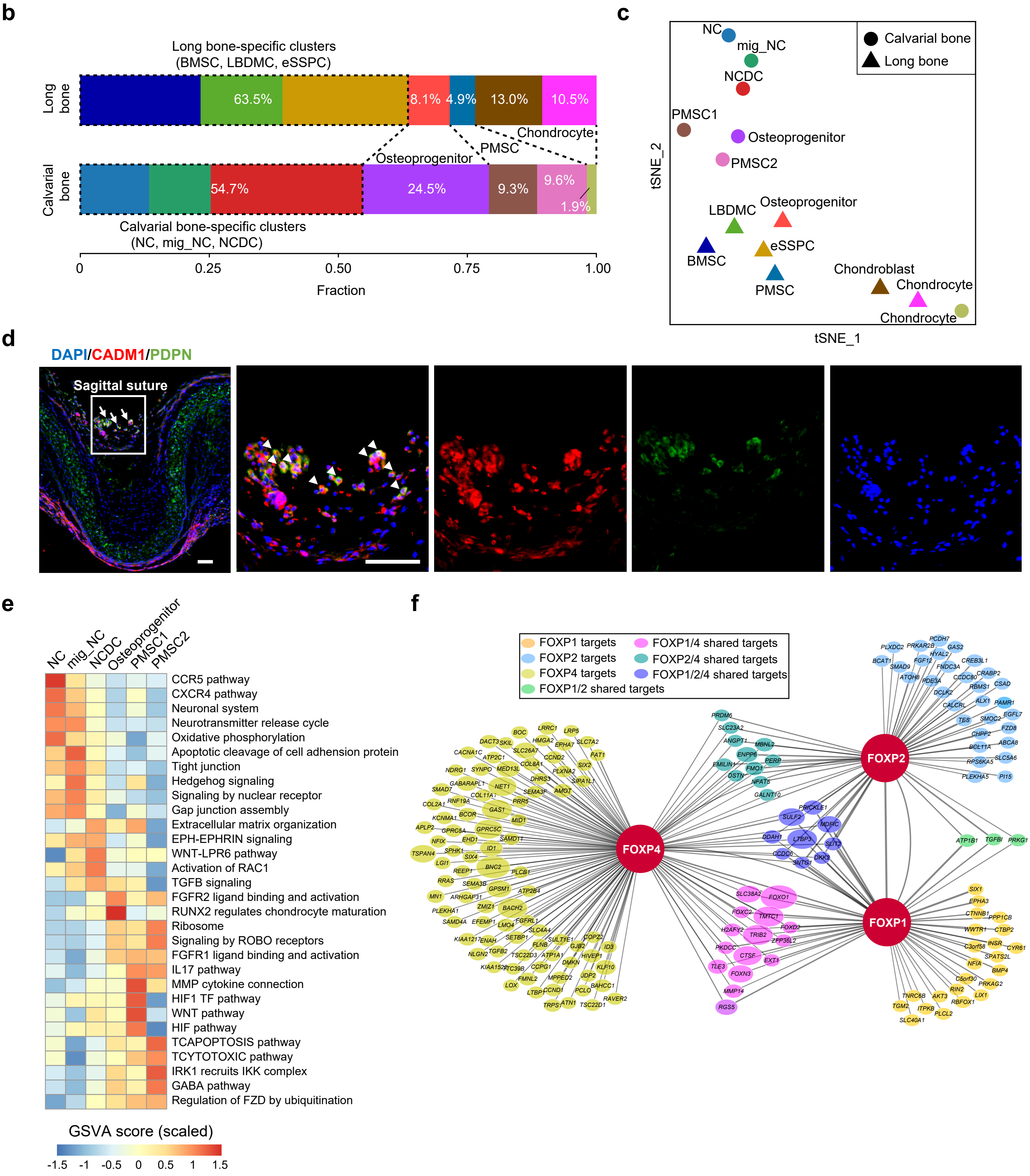

Supplementary Fig. 6. Further characterizations of human embryonic calvaria

a, Table summary of the 8 WPC human embryonic calvarial bone samples for scRNA-seq and immunostaining.

b, Stacked bar charts comparing the distribution of 8 WPC long bone and calvarial subsets. Dashed boxes indicated skeletal site-specific clusters. The three shared clusters (osteoprogenitor, PMSC and chondrocyte) were highlighted by dash lines.

c, t-distributed stochastic neighbor embedding(t-SNE) projection of indicated subsets from long bones and calvarial bones to compare the transcriptomic similarities at the pseudo-bulk level.

d, Immunofluorescent images of $PDPN^+CADM1^+$ cells in 8 WPC human calvarial bones. Snapshot image of the calvarial region surrounding sagittal suture was shown on the left. $PDPN^+CADM1^+$ cells (arrows) were found in the outer layer of sagittal mesenchyme. Arrow heads indicated enlarged $PDPN^+CADM1^+$ cells. Merged and single-channel images of DAPI (blue), CADM1 (red) and PDPN (green) were shown (n=2 embryos). Scale bars: 100 μm.

e, Heatmap showing pathways differentially enriched in calvarial bone subsets by GSVA, colored by scaled mean of GSVA scores.

f, The FOXP1/2/4 regulon network in 8 WPC human calvarial bone subsets. Line thickness indicated the level of GENIE3 weights. Dot size indicated the number of enriched TF-motif.
